# Supplementary material for: Understanding preferences for behaviour change support as part of the NHS Health Check: a qualitative study with adults from underserved minoritised ethnic communities
Source: BMJ Open. 2026 Jan 14;16(1):e111413. doi: 10.1136/bmjopen-2025-111413 (PMC12820816; doi:10.1136/bmjopen-2025-111413)
Supplement: Supplementary data [file bmjopen-16-1-s001.pdf]

Supplementary File

Supplementary Material S1: Screenshots of the Digital Prototype (Final Version)

Note: The prototype signposts to real organisations (e.g., Slimming World), which are publicly available. All personal details and cardiovascular risk information shown in this prototype are fictitious; no real participant data were used.

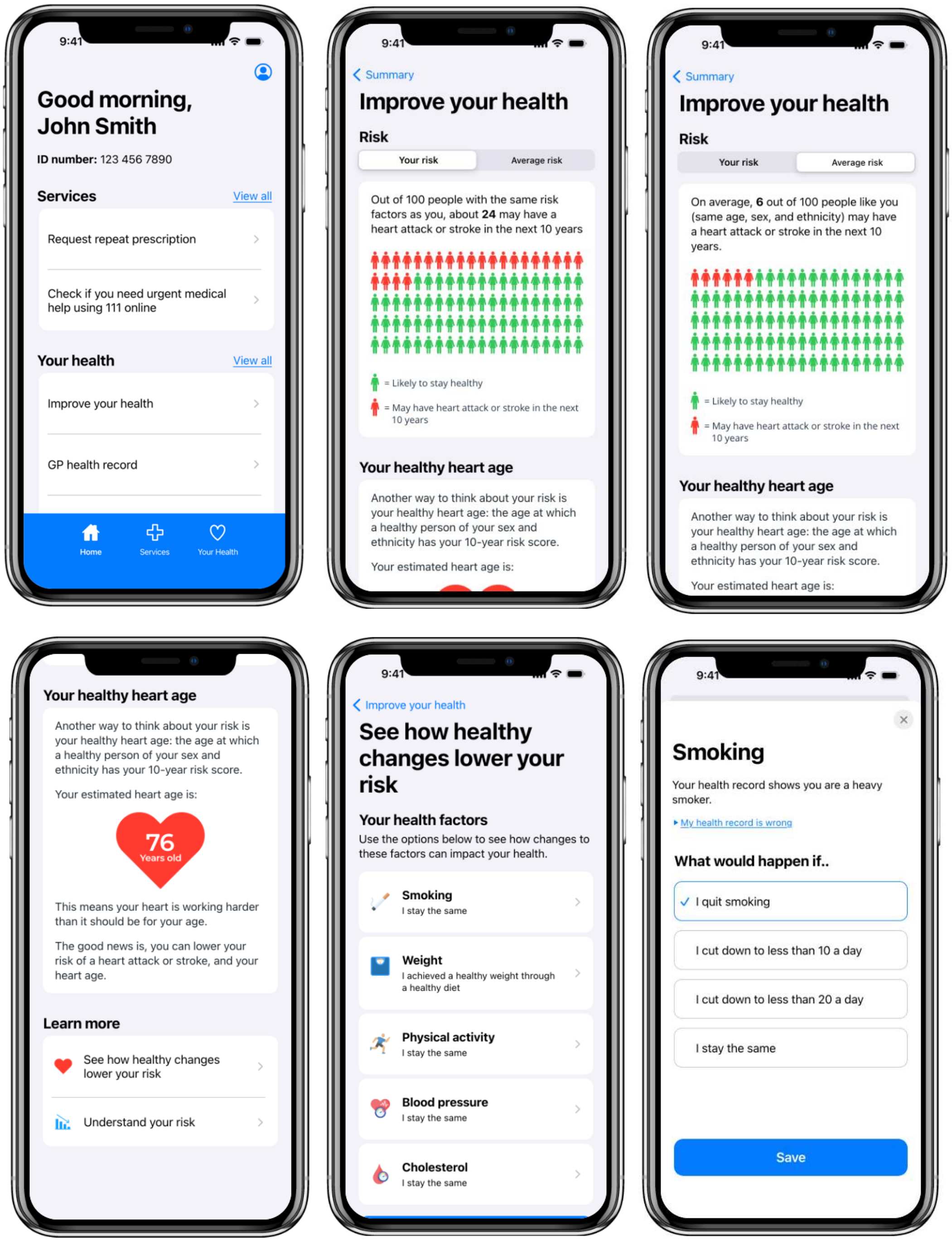

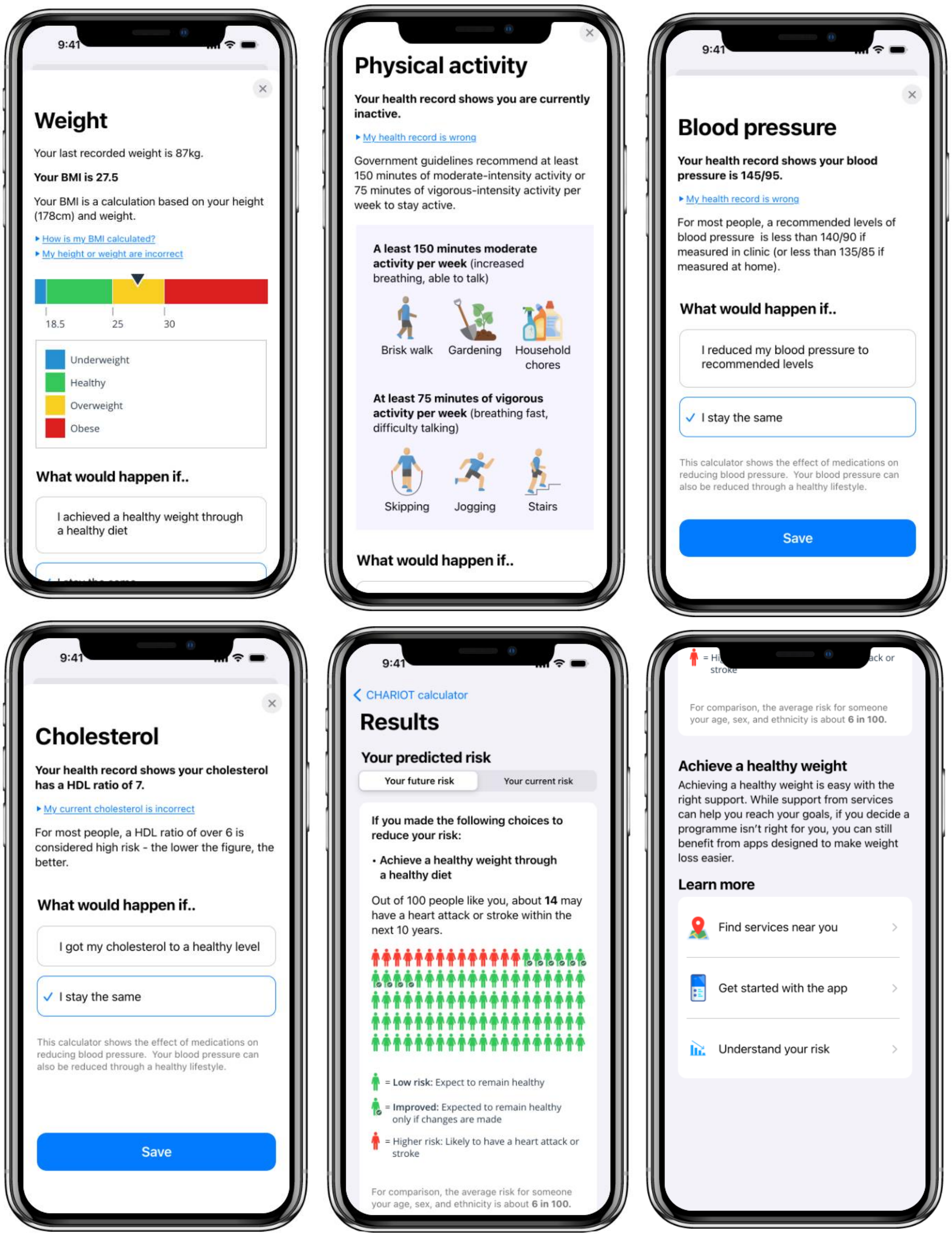
Weight
Your last recorded weight is 87kg.
Your BMI is 27.5
Your BMI is a calculation based on your height (178cm) and weight.
How is my BMI calculated?
My height or weight are incorrect
18.5
25
30
Underweight
Healthy
Overweight
Obese
What would happen if..
I achieved a healthy weight through a healthy diet
Physical activity
Your health record shows you are currently inactive.
My health record is wrong
Government guidelines recommend at least 150 minutes of moderate-intensity activity or 75 minutes of vigorous-intensity activity per week to stay active.
A least 150 minutes moderate activity per week (increased breathing, able to talk)
Brisk walk
Gardening
Household chores
At least 75 minutes of vigorous activity per week (breathing fast, difficulty talking)
Skipping
Jogging
Stairs
What would happen if..
Blood pressure
Your health record shows your blood pressure is 145/95.
My health record is wrong
For most people, a recommended levels of blood pressure is less than 140/90 if measured in clinic (or less than 135/85 if measured at home).
What would happen if..
I reduced my blood pressure to recommended levels
I stay the same
This calculator shows the effect of medications on reducing blood pressure. Your blood pressure can also be reduced through a healthy lifestyle.
Save
Cholesterol
Your health record shows your cholesterol has a HDL ratio of 7.
My current cholesterol is incorrect
For most people, a HDL ratio of over 6 is considered high risk - the lower the figure, the better.
What would happen if..
I got my cholesterol to a healthy level
I stay the same
This calculator shows the effect of medications on reducing blood pressure. Your blood pressure can also be reduced through a healthy lifestyle.
Save
CHARIOT calculator
Results
Your predicted risk
Your future risk
Your current risk
If you made the following choices to reduce your risk:
• Achieve a healthy weight through a healthy diet
Out of 100 people like you, about 14 may have a heart attack or stroke within the next 10 years.
= Low risk: Expect to remain healthy
= Improved: Expected to remain healthy only if changes are made
= Higher risk: Likely to have a heart attack or stroke
For comparison, the average risk for someone your age, sex, and ethnicity is about 6 in 100.
For comparison, the average risk for someone your age, sex, and ethnicity is about 6 in 100.
Achieve a healthy weight
Achieving a healthy weight is easy with the right support. While support from services can help you reach your goals, if you decide a programme isn't right for you, you can still benefit from apps designed to make weight loss easier.
Learn more
Find services near you
Get started with the app
Understand your risk

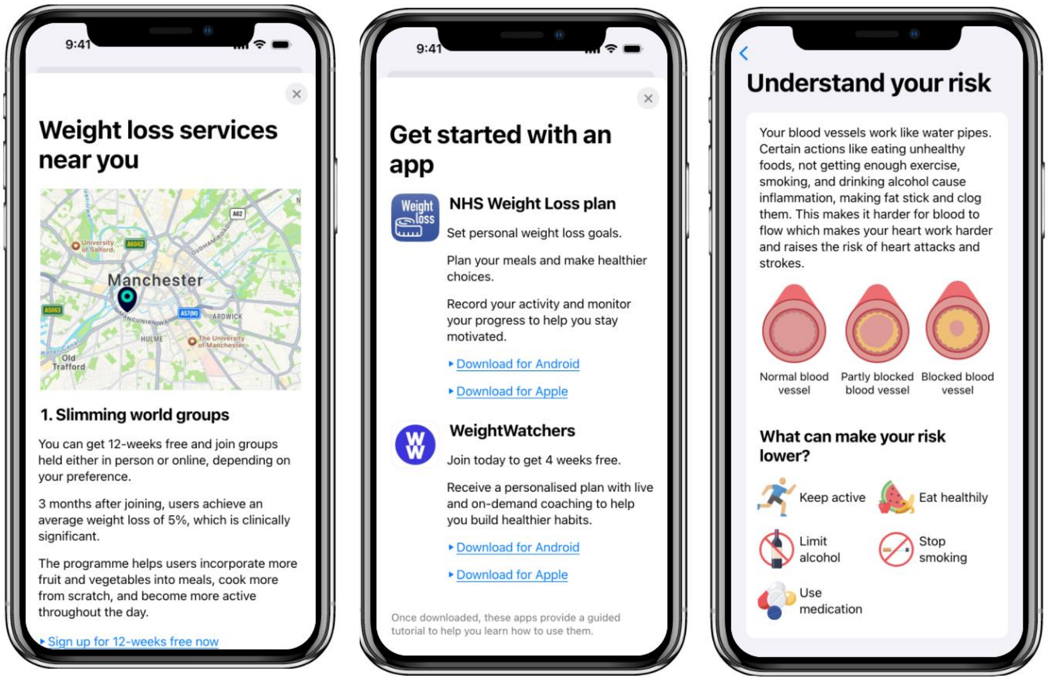
9:41
Weight loss services near you
Manchester
1. Slimming world groups
You can get 12-weeks free and join groups held either in person or online, depending on your preference.
3 months after joining, users achieve an average weight loss of 5%, which is clinically significant.
The programme helps users incorporate more fruit and vegetables into meals, cook more from scratch, and become more active throughout the day.
Sign up for 12-weeks free now
9:41
Get started with an app
NHS Weight Loss plan
Set personal weight loss goals.
Plan your meals and make healthier choices.
Record your activity and monitor your progress to help you stay motivated.
Download for Android
Download for Apple
WeightWatchers
Join today to get 4 weeks free.
Receive a personalised plan with live and on-demand coaching to help you build healthier habits.
Download for Android
Download for Apple
Once downloaded, these apps provide a guided tutorial to help you learn how to use them.
Understand your risk
Your blood vessels work like water pipes. Certain actions like eating unhealthy foods, not getting enough exercise, smoking, and drinking alcohol cause inflammation, making fat stick and clog them. This makes it harder for blood to flow which makes your heart work harder and raises the risk of heart attacks and strokes.
Normal blood vessel
Partly blocked blood vessel
Blocked blood vessel
What can make your risk lower?
Keep active
Eat healthily
Limit alcohol
Stop smoking
Use medication

Supplementary Material S2: Focus Group and Interview Topic Guides

These topic guides were used across the wider qualitative study. Not all questions contributed data to the analysis reported in the accompanying manuscript; only the relevant sections of the dataset were analysed for the manuscript's specific aims.

1. Focus group/ workshop schedule

Introduction and housekeeping

- Welcome participants, thank them for attending, explain ground rules.
- Explain the purpose of the session and its role within the wider study.
- Introduce the research team and invite participants to introduce themselves.
- Emphasise that all contributions are confidential and that participants should respect each other's privacy.
- Explain that all data will be anonymised during transcription and reporting; no identifiable information will be included in publications.
- Explore participants' existing understanding of cardiovascular disease (CVD) and familiarity with NHS Health Checks before explaining context for the prototype.
- Show the following prototype presentation (1.1-1.7).

Prototype presentation

1.1 Navigation page

- A discussion about how to navigate to the calculator
- Consider what language/labels feel clear and accessible.

1.2 Text explanation of risk calculator

- A discussion about whether people understand text information explaining risk calculator, and if any other information is needed to support their understanding.

1.3 Risk score result and visual display

- A discussion around presentation of risk results, including icon arrays, colours used.

1.4 Heart age result display

Griffiths S, *et al. BMJ Open* 2026; 16:e111413. doi: 10.1136/bmjopen-2025-111413

- A discussion about presentation of Heart Age and comparative risks.
- Explore whether participants find this combination of information helpful for understanding cardiovascular risk.

1.5 Risk explanation

- Gather views on how risk information is presented, if it is understood, and views on the analogy used.
- Discuss what would make the information most understandable and meaningful.

1.6 Risk manipulation

- Explore views on seeing potential reductions in risk through health behaiour changes (stopping smoking, losing weight, increasing physical activity, reducing blood pressure, improving cholesterol).
- Discuss whether the behaviour change options presented are perceived as feasible or motivating.
- Explore how participants would like reductions in risk to be presented (use of risk bar, icon array, and whether the green icons with ticks used to show the difference between current and potential risk are clear).

1.7 Behaviour change support signposting

- Explore views on the example of how a hypothetical user could be signposted to resources to help them change CVD risk factors (e.g. smoking cessation services, weight loss services, information on how they could reduce their blood pressure) etc.
- Explore views on how helpful this concept would be, what could be improved/ made more clear or useful.

Closing discussion

- Invite participants to share any additional thoughts or reflections.
- Thank participants for their contributions.
- Remind participants they can contact the research team if they have follow-up questions or concerns.

Griffiths S, *et al. BMJ Open* 2026; 16:e111413. doi: 10.1136/bmjopen-2025-111413

2. Interview Topic Guide

1. Was there anything about the risk score or information that felt confusing or unclear to you?
2. What, if anything, was memorable?

Prompt: Are there any particular features you think would encourage you to use this app?

3. At the beginning it tells you your risk score. Would seeing your risk presented like this influence you in any way, in terms of making healthier changes?
4. Do you think you would like to look back at your risk score over time, to see how it changes if you went away and made healthier changes?
5. Could you see yourself using this tool independently, or do you think you'd need to be shown it by a professional first?
6. Is there anything other features you would like to be included, that would make the app more appealing?

Prompt: How would you feel about there being techniques to help you manage making healthier changes, e.g., goal setting?

7. Is there anything that isn't on there that you would have liked to learn about, related to heart health?
8. Did you leave with the impression from the app that healthier changes could make a difference to heart health?

Prompt: or cholesterol, blood pressure?

4. There was an example of how grease builds up in pipes used to explain what goes on inside your blood vessels- how did you find that?

Prompt: How did it make you feel towards making healthy or unhealthy choices?

10. If you were thinking about improving your heart health, are there any challenges that come to mind that the app, or other support, might be able to help with?

Prompt: Are there any other ways you'd prefer to see this information, e.g., videos?

If videos: Would you be interested in seeing animations or real people in videos?

5. There were weight loss programmes listed on the app as options for support. The idea would be the same for any other goal that people wanted to achieve with their health where that kind of support is available. Do you think you would make the leap to signing up to programmes like that to receive support?
12. Is there anything about a programme that would make going more or less appealing to you? *Prompts: Who runs it, what age range it is targeted for.*
13. Are you more interested doing things in the community in person, or would you access support online – why?
14. How would you describe your confidence in terms of going on to make healthier changes after receiving this kind of information?

Prompt: What kind of support would help you feel more confident about making and sticking to healthier changes?

15. Is there anything else you wanted to add - any final thoughts?

Supplementary Material Table S3: SRQR Checklist

Standards for Reporting Qualitative Research (SRQR): a synthesis of recommendations

All items of this checklist are directly cited from O'Brien BC, Harris IB, Beckman TJ, Reed DA, Cook DA.

Standards for reporting qualitative research: A synthesis of recommendations. *Academic Medicine*.

2014;89(9):1245-1251. doi:10.1097/ACM.0000000000000388

	Item Description	Location (or reason for not reporting)
**Title & Abstract**		
Title	Describe the nature and topic of the study. Identify the study as qualitative or indicate the approach or data collection methods.	Title page
Abstract	Summarise the key elements of the study using the abstract format of the intended publication.	Title page
**Introduction**		
Problem Formulation	Describe the problem/phenomenon studied, its significance, relevant theory and empirical work, and gaps in current knowledge.	Introduction; paragraphs 1, 2, 3, 4
Purpose or research question	Describe the purpose of the study and specific objectives or questions.	Introduction; paragraph 4. Aim
**Methods**		
Qualitative approach and research paradigm	Describe your qualitative approach, your guiding theory (if appropriate), and research paradigm, and reasons for your choices.	Methods, Study design, Analysis
Researcher characteristics and reflexivity	Describe how researchers' characteristics may influence the research, including personal attributes, qualifications/experience, relationship with participants, assumptions, and/or presuppositions; potential or actual interaction between researchers' characteristics and the research questions, approach, methods, results and/or transferability.	Methods, Analysis; paragraph 2
Context	Describe the setting/site(s) in which the study was conducted, why it was selected, and any other salient contextual factors that may influence the study.	Methods, Participant recruitment; paragraph 1
Sampling strategy	Describe how and why research participants, documents, or events were selected; criteria for deciding when no further sampling was necessary, and the rationale for those criteria.	Methods, Participant recruitment; paragraph 2
Ethical issues pertaining to human subjects	Describe any approval by an appropriate ethics review board and participant consent, or explain any lack thereof. Describe any other confidentiality and data security issues.	Methods, Study design
Data collection methods	Describe the types of data collected; details of data collection procedures including (as appropriate) start and stop dates of data collection and analysis, iterative process, triangulation of sources/methods, and modification of procedures in response to evolving study findings. Describe your rationale for these choices.	Methods, Data collection

Data collection instruments and technologies	Describe any instruments (e.g., interview guides, questionnaires) and devices (e.g., audio recorders) used for data collection; describe if/how the instrument(s) changed over the course of the study.	Methods, Materials
Units of study	Describe the number and relevant characteristics of participants, documents, or events included in the study. Describe the level of participation.	Results; paragraph , Table 1
Data processing	Describe the methods for processing data prior to and during analysis, including transcription, data entry, data management and security, verification of data integrity, data coding, and anonymisation / deidentification of excerpts.	Methods, Analysis; paragraph 1
Data analysis	Describe the process by which inferences, themes, etc. were identified and developed, including the researchers involved in data analysis; usually references a specific paradigm or approach. Describe why you chose this process.	Methods, Analysis; paragraph 1, 2
Techniques to enhance trustworthiness	Describe any techniques to enhance trustworthiness and credibility of data analysis,(e.g., member checking, triangulation, audit trail). Describe why you chose these techniques.	Methods, Analysis; paragraph 2
**Results**		
Synthesis and interpretation	Describe the main findings (e.g., interpretations, inferences, and themes); might include development of a theory or model, or integration with prior research or theory.	Results; Table 2. & First paragraph beneath each theme heading.
Links to empirical data	Provide evidence (e.g., quotes, field notes, text excerpts, photographs) to substantiate analytic findings.	Results; embedded throughout (e.g., paragraph 4).
**Discussion**		
Integration with prior work, implications, transferability, and contribution(s) to the field	Summarize the main findings, explain how findings and conclusions connect to, support, elaborate on, or challenge conclusions of earlier scholarship; discuss the scope of application/generalizability; identify unique contribution(s) to scholarship in a discipline or field.	Discussion; paragraph 1-10
Limitations	Discuss the trustworthiness and limitations of findings	Discussion; paragraph 7
**Other**		
Conflicts of interest	Describe any potential sources of influence or perceived influence on study conduct and conclusions. Describe how these were managed.	Declarations, Competing interests
Funding	Describe sources of funding and other support. Describe the role of funders in data collection, interpretation, and reporting.	Declarations, Funding

Griffiths S, *et al. BMJ Open* 2026; 16:e111413. doi: 10.1136/bmjopen-2025-111413
