# Supplementary material for: Understanding preferences for behaviour change support as part of the NHS Health Check: a qualitative study with adults from underserved minoritised ethnic communities
Source: BMJ Open. 2026 Jan 14;16(1):e111413. doi: 10.1136/bmjopen-2025-111413 (PMC12820816; doi:10.1136/bmjopen-2025-111413)
Supplement: Author's manuscript [file bmjopen-2025-111413.draft_revisions.pdf]

BMJ Open

BMJ Open is committed to open peer review. As part of this commitment we make the peer review history of every article we publish publicly available.

When an article is published we post the peer reviewers' comments and the authors' responses online. We also post the versions of the paper that were used during peer review. These are the versions that the peer review comments apply to.

The versions of the paper that follow are the versions that were submitted during the peer review process. They are not the versions of record or the final published versions. They should not be cited or distributed as the published version of this manuscript.

BMJ Open is an open access journal and the full, final, typeset and author-corrected version of record of the manuscript is available on our site with no access controls, subscription charges or pay-per-view fees (http://bmjopen.bmj.com).

If you have any questions on BMJ Open's open peer review process please email info.bmjopen@bmj.com

BMJ Open

Understanding Preferences for Behaviour Change Support as part of the NHS Health Check: A Qualitative Study with Adults from Underserved Minoritised Ethnic Communities

Journal:	*BMJ Open*
Manuscript ID	bmjopen-2025-111413
Article Type:	Original research
Date Submitted by the Author:	24-Sep-2025
Complete List of Authors:	Griffiths, Sophie; The University of Manchester School of Health Sciences, Centre for Health Psychology<br>Bartlett, Yvonne Kiera; The University of Manchester School of Health Sciences, Centre for Health Psychology<br>French, David; The University of Manchester School of Health Sciences, Centre for Health Psychology<br>McMillan, Brian; The University of Manchester School of Health Sciences, Centre for Primary Care and Health Services Research
Keywords:	Health Services, PUBLIC HEALTH, QUALITATIVE RESEARCH, Preventive Health Services, Patient Preference, Health Equity

SCHOLARONE™
Manuscripts

I, the Submitting Author has the right to grant and does grant on behalf of all authors of the Work (as defined in the below author licence), an exclusive licence and/or a non-exclusive licence for contributions from authors who are: i) UK Crown employees; ii) where BMJ has agreed a CC-BY licence shall apply, and/or iii) in accordance with the terms applicable for US Federal Government officers or employees acting as part of their official duties; on a worldwide, perpetual, irrevocable, royalty-free basis to BMJ Publishing Group Ltd ("BMJ") its licensees and where the relevant Journal is co-owned by BMJ to the co-owners of the Journal, to publish the Work in this journal and any other BMJ products and to exploit all rights, as set out in our licence.

The Submitting Author accepts and understands that any supply made under these terms is made by BMJ to the Submitting Author unless you are acting as an employee on behalf of your employer or a postgraduate student of an affiliated institution which is paying any applicable article publishing charge ("APC") for Open Access articles. Where the Submitting Author wishes to make the Work available on an Open Access basis (and intends to pay the relevant APC), the terms of reuse of such Open Access shall be governed by a Creative Commons licence – details of these licences and which Creative Commons licence will apply to this Work are set out in our licence referred to above.

Other than as permitted in any relevant BMJ Author's Self Archiving Policies, I confirm this Work has not been accepted for publication elsewhere, is not being considered for publication elsewhere and does not duplicate material already published. I confirm all authors consent to publication of this Work and authorise the granting of this licence.

For peer review only

Understanding Preferences for Behaviour Change Support as part of the NHS Health Check: A Qualitative Study with Adults from Underserved Minoritised Ethnic Communities

Authors

Sophie Griffiths[1]*, Dr Y Kiera Bartlett[1], Prof David P French[1], Dr Brian McMillan[2]

*Corresponding author: Sophie Griffiths, Coupland Building 1, Manchester, M15 6FH.

sophie.griffiths@manchester.ac.uk, 0161 306 9078

Affiliations

[1]Centre for Health Psychology, Manchester, England, [2]Centre for Primary Care and Health Services Research, Manchester, England.

Email addresses

Sophie Griffiths sophie.griffiths@manchester.ac.uk

Y Kiera Bartlett kiera.bartlett@manchester.ac.uk

David P French david.french@manchester.ac.uk

Brian McMillan brian.mcmillan@manchester.ac.uk

Keywords

Preventive Health Services; Qualitative Research; Public Health; Patient Preferences; Health Equity

Word count

3972/ 4000

For peer review only - http://bmjopen.bmj.com/site/about/guidelines.xhtml

Abstract (Word count: 300/ 300)

Introduction: NHS Health Checks provide individuals with cardiovascular disease risk scores alongside advice and signposting to behaviour change support. A particular problem is that the support people receive is often poorly delivered, absent, or not tailored to the needs of people in deprived communities, which risks exacerbating health inequalities. Improving this support is critical if NHS Health Checks are to achieve their goals of prevention and equity.

Objectives: To explore needs for and preferences in behaviour change support among adults in deprived areas, using a digital prototype presenting cardiovascular disease risk information and signposting to services.

Design: A longitudinal qualitative study involving focus groups and semi-structured follow-up interviews.

Participants and setting: Adults from minoritised ethnic groups eligible for NHS Health Checks, recruited online and through a community centre, with both methods targeting high-deprivation areas.

Method: Participants were first shown the digital prototype in focus groups to generate discussion. Follow-up interviews captured more in-depth reflections on needs for behaviour change support. Data were analysed using reflexive thematic analysis.

Results: We conducted four focus groups and 20 follow-up interviews with 23 adults who identified as being from minoritised ethnic backgrounds. We developed three themes: (1) Trusted information to counter confusion and misinformation; (2) Support that makes change feel possible and meaningful, through culturally and personally relevant advice that addresses unhelpful beliefs about risk reduction and behaviour change; and (3) Ensuring access to inclusive, socially connected environments that feel supportive and conducive to action.

Conclusions: For minoritised ethnic adults in deprived areas, NHS Health Check support should build on everyday practices and foster positive perceptions of services. Alongside service-level changes, policy action is needed to remove structural barriers (e.g., cost, safety) that limit people's

For peer review only

ability to act on advice. Such changes could enhance the programme's contribution to reducing inequalities in cardiovascular disease prevention.

Article summary

Strengths and limitations of this study

- This study addresses a key gap in the NHS Health Check literature by exploring the support needs of participants from minoritised ethnic communities in deprived areas, a group rarely represented despite heightened cardiovascular risk.
- A digital prototype, presenting hypothetical CVD risk in a realistic NHS Health Check format, was used to explore preferences for support.
- Focus groups facilitated discussion of support needs, with follow-up interviews capturing subsequent reflections and providing additional depth.

For peer review only

For peer review only - http://bmjopen.bmj.com/site/about/guidelines.xhtml

Introduction

Cardiovascular disease (CVD) is the leading cause of death worldwide, responsible for nearly one-third of all annual deaths[1]. In the UK, over 7.6 million people live with CVD[2]. Modifiable conditions such as high cholesterol, high blood pressure, and type 2 diabetes increase CVD risk and are strongly influenced by social and economic determinants including deprivation[3]. Effective prevention therefore requires engaging people whose health behaviours are shaped by intersecting social and economic pressures.

The NHS Health Check (NHSHC) invites adults aged 40–74 years without established CVD to assess and manage their risk. Uptake is lowest in communities with the greatest burden: socioeconomically deprived neighbourhoods and minoritised ethnic groups[4]. To improve reach, commissioners have extended delivery beyond general practice into community venues such as pharmacies, mosques, and pop-up clinics, which tend to attract higher-risk and underserved groups.

Attendance alone, however, does not guarantee improved cardiovascular outcomes. Benefits depend on whether people understand and act on the advice received. While policy and research have focused on increasing uptake, less attention has been given to the equally critical step of supporting behaviour change. The generic advice often provided in NHSHCs[5] may be particularly ineffective for those facing combined socioeconomic and ethnic disadvantage. Follow-up support also appears under-utilised: a pharmacy-based study found low uptake of recommended follow-up, representing a missed opportunity to assist those most in need[6].

Prior qualitative research in a socioeconomically deprived area in south England concluded that CVD prevention support should be co-designed with local organisations, delivered by trusted community members, and centred on peer bonds and enjoyment rather than generic health messages[7]. However, that study explored general preventative preferences rather than needs formed after receiving CVD risk information and did not consider how ethnicity and deprivation intersect to compound disadvantage. To address these gaps, we showed adults from minoritised ethnic and socioeconomically deprived backgrounds a digital prototype presenting hypothetical CVD risk

For peer review only - http://bmjopen.bmj.com/site/about/guidelines.xhtml

information and support options, similar to the format used in the digital NHSHC pilot[8], providing a realistic prompt for discussing their support needs.

Aim

To identify needs and preferences for behaviour change support among adults from socioeconomically deprived and minoritised ethnic backgrounds, using a digital prototype to ground discussions in the context of NHSHCs.

Method

Study design

This longitudinal qualitative study used focus groups and follow-up one-to-one semi-structured interviews. The study received University of Manchester Research Ethics approval (2024-19916-37529). All participants provided written or electronic consent.

Participant recruitment

Participants were recruited through in-person and online approaches targeting socioeconomically deprived areas. Deprivation was assessed using the English Index of Multiple Deprivation (IMD), which ranks individual's postcodes in deciles range from 1 (most deprived) to 10 (least deprived) based on multiple factors including income and employment. The community centre used for in-person recruitment was in Greater Manchester, in an area ranked in the most deprived IMD decile (1) and primarily served South Asian communities. Additional recruitment took place via social media, focusing on high-deprivation areas of Greater Manchester.

Eligibility criteria were the same as those for the NHSHC, namely adults aged 40 to 74 years old with no previous diagnosis of CVD. Participants were recruited until sufficient information power was achieved[9], meaning that the data collected were rich and relevant enough to adequately address the research questions.

For peer review only

Data collection

Focus groups explored initial responses to the digital prototype, beginning in October 2024 and ending in April 2025. Participants were told that in practice, risk information would be linked to electronic health records. Discussions covered understanding of CVD risk, attitudes towards behaviour change, perceptions of signposting, and preferred support types. A short demographic questionnaire was completed at the end of each group.

Participants were invited to a one-to-one follow-up interview at least one week later to reflect on the prototype and discuss personal needs and preferences in greater depth. Interviews were held via Zoom or at the community centre. An interpreter was available as needed during focus groups and interviews. The prototype was iterated between each focus group in line with participant feedback.

Materials

We used questionnaires (Qualtrics XM or paper) to collect demographic information: age (years), gender, and ethnicity (2011 England and Wales Census categories), highest education qualification, and full postcode.

The digital prototype of an app was developed by a specialist Digital Health Software Team based at the University of Manchester[10] using Marvel software[11]. The first iteration was informed by patient and public involvement (PPI), literature on risk communication[12-14], and psychological theory[15,16]. Each iteration was refined through a separate co-design process, with details described in a related study. It included visual CVD risk displays alongside a simulated CVD risk estimation tool, illustrating potential risk reductions from health behaviour changes based on a cardiovascular risk prediction algorithm currently under development[17], as well as brief cardiovascular health education (including national physical activity guidelines) and referral/ signposting to weight management services and digital tools (see Figure 1 and Supplement A).

Figure 1. Screenshots of the digital prototype presenting CVD risk information and signposting to behaviour change support.

For peer review only - http://bmjopen.bmj.com/site/about/guidelines.xhtml

A workshop schedule guided participants through the prototype and facilitated discussions specific to the prototype. Focus group and interview schedules were reviewed and refined after the initial subset of participants.

Patient and public involvement

We involved PPI members from the development stage to help shape the focus and priorities of this research[18]. All participant-facing documents were reviewed to enhance accessibility. In July 2024, an online session with three PPI members informed the first prototype iteration.

Analysis

Focus groups and interviews were audio-recorded, transcribed verbatim, and anonymised. Transcripts were analysed using reflexive thematic analysis[19]. Initial coding focused on semantic codes, capturing explicit statements, while later stages of analysis became more interpretative, using latent codes to explore underlying meanings and contextual influences on participants' accounts.

Reflexivity was maintained throughout, with SG regularly discussing interpretations with YKB to enhance rigour. SG's identity as a white woman may have shaped interactions with participants - most of whom were also women. This shared gender identity may have built rapport and encouraged participants to share more openly. While SG's outsider perspective might have limited the full appreciation of cultural nuances, it also encouraged a more open approach to clarifying participants' perspectives and avoiding assumptions. This outsider status may have prompted participants to elaborate more fully in their responses, providing richer data for analysis.

This manuscript was prepared in line with the Standards for Reporting Qualitative Research (SRQR) guideline[20] and checklist[21], provided in Supplement B.

Results

A total of 23 participants attended four focus groups: with 20 completing follow-up interviews. Six were recruited online and 17 via a community centre. Ages ranged from 40–74 years (median 61). Five identified as male and 18 as female. All were of Asian or Asian British ethnicity, except one who

For peer review only - http://bmjopen.bmj.com/site/about/guidelines.xhtml

identified as being of Arab ethnicity. Most (n = 19/23, 82.6%) lived in areas ranked IMD deciles 1-4. Many participants held educational qualifications which did not directly align with UK qualification frameworks (e.g. from Pakistan). An interpreter assisted with translation for six participants.

Three themes with sub-themes were produced and are outlined in Table 1.

Table 1. Summary of the themes and sub-themes identified in this study

Themes	Sub-themes
Trusted information as a foundation for engagement	Navigating health misinformation
	Trust in tailored advice
Making change feel manageable and worthwhile	Acknowledgement of everyday efforts and priorities
	The toll of guilt, regret, and rigid rules
	Working with cultural values
Creating environments where change feels possible	Safe, inclusive, and accessible environments
	The motivating power of human connection

Trusted information as a foundation for engagement

This theme captures participants' need for clear and credible information, linking concerns about widespread misinformation with the desire for personalised advice from trusted sources.

Navigating health misinformation

Participants reported needing clear, trustworthy health guidance for their health behaviours. Many felt overwhelmed or paralysed by the sheer volume of conflicting advice, particularly when its credibility was uncertain or when they had difficulty securing GP appointments.

For peer review only - http://bmjopen.bmj.com/site/about/guidelines.xhtml

For peer review only

'Let's say you're trying to get healthy. There's so many people, like influencers that give you the wrong information… You feel overwhelmed. You just want to know the truth from someone.' Nadia, 44 years.

Information overload was not confined to the internet; contradictory tips exchanged among friends and family also amplified uncertainty.

'She doesn't follow online; she doesn't trust them at all… From her friends she gets suggestions like, 'Do not eat in the morning', 'Change your flour', 'Do some more walk', 'Do this, do that'—it's sometimes too much, and she's quite confused what she needs to do… That's why she wants two-three simple things from an expert that she could follow.' Lubna via interpreter, 59 years.

The lack of accessible, authoritative advice meant navigating multiple, often inconsistent information sources. In response, participants voiced a clear preference for brief, expert-endorsed guidance to bypass contradictory messages and give them a manageable starting point for behaviour change.

Trust in tailored advice

Participants valued that the information within the prototype carried the authority of a trusted institution and offered recommendations that felt specific to their own circumstances. Having a single, reliable point of reference spared them the effort of searching online and turned uncertainty into a clear, personalised next step to act on.

'Some people just don't know what to do. Like you said, if you put on there [the app] guidelines, 'Try this, do this, this', you've got something to look at and then do it. Because if there's nothing to guide you… Nobody's going to look on YouTube or Google… But if it's on your app, your own health app, they'll say, 'Oh this is good for me, I'll try this'. Because it's all about you, and nobody else.' Tahira, 54 years.

For peer review only - http://bmjopen.bmj.com/site/about/guidelines.xhtml

Personalised advice mattered not only for its practicality but for the relief it brought. When participants felt as though guidance had been written for them, it meant not having to follow generic, one-size-fits-all guidance, which could be overwhelming and detrimental to mental wellbeing.

> *'Something for you is not for something for someone else, isn't it? Because not everybody's the same… sometimes it's for the greater good of your mental health as well because you don't want to end up following too many things and become depressed.'* Nadia, 44 years.

Participants framed trusted, personalised guidance as both more credible and more emotionally manageable, giving them a clear direction in an otherwise confusing information landscape.

Making change feel manageable and worthwhile

This theme highlights participants' desire for advice relevant to their daily lives, while noting that unhelpful beliefs and cultural context could undermine its actionability if not addressed.

Acknowledgement of everyday efforts and priorities

Participants described how standard recommendations felt disconnected from their daily lives. They questioned whether informal and incidental forms of movement embedded in their daily routines counted towards meeting the physical activity recommendations.

> *'Even carrying the basket up and down the stairs… you don't realise it, how many times you go up and down.'* Zara, 41 years.

Recognising these small, everyday efforts could help motivate change and reduce discouragement, particularly when broader life pressures, like mental wellbeing, disabilities, cost of living, or caring responsibilities, shaped what felt possible to attempt.

> *'You'd have to physically get a membership for the swim and cycle, like you have to purchase something… You could do stuff in the house and you're still using energy. But like obviously with cost of living… I can do a walk, a run, the stairs.'* Nadia, 44 years.

For peer review only - http://bmjopen.bmj.com/site/about/guidelines.xhtml

Cultural traditions were an added layer of context shaping what felt manageable and sustainable. For example, foods shared during celebrations were often high in fat and sugar, making dietary changes feel incompatible with cultural norms and practices. Participants were open to learning culturally relevant alternatives to make healthy eating feel more realistic without having to abandon traditions.

The toll of guilt, regret, and rigid rules

The prospect of adopting healthier behaviour*s* often carried emotional weight due to participants' beliefs about CVD risk and behaviour change. Some expressed fatalism, the belief that serious illness was inevitable regardless of current efforts. Others reflected on past choices with regret, expressing a sense that the damage to their health had already been done.

> *'If only we'd known all this when we were kids... we were eating all junk. But now... we realise, 'If only I had done that'.'* Tahira, 54 years.

Others described an all‐or‐nothing mindset, where a single lapse, such as eating unhealthy food, was seen as 'cheating' - a moral framing that imposed pressure and reinforced feelings of failure. Unhealthy eating was perceived to negate other efforts, such as exercise, despite cardiovascular benefits beyond weight loss.

> *'When I'm in any exercise session… in my head I'm telling myself I have to eat healthy. I can't cheat or anything. There's no point stretching if I'm going to go home and start eating rubbish.'* Mahila, 65 years.

When prompted to discuss goal setting, participants often expressed scepticism. Setting goals was seen as overwhelming, particularly when goals were misaligned with current capabilities.

> *'You can't go from zero to 10 by goal setting. Sometimes it has the opposite effect for me… I prefer just doing natural things that feel comfortable for me… it's kind of like Slimming World, it creates a bit of like 'oh, am I a failure?'… Rather than if you just said 'Oh, you did 2000 last week, that's fantastic. Try to do 10 more'.'* Nadia, 44 years.

For peer review only - http://bmjopen.bmj.com/site/about/guidelines.xhtml

Participants also described beliefs about diet and activity that made behaviour change feel unnecessarily difficult. Some of these reflected oversimplified or inaccurate understandings of what needed to be restricted.

> *'Chapati, rice — they make you fat.'* Rubina, 74 years.

Such beliefs could lead to cognitively demanding restrictions that were taxing yet of limited benefit. However, more structured approaches, like calorie counting, which might theoretically reduce the need to eliminate foods entirely, were still overwhelming for some participants.

Working with cultural values

Although the prototype emphasised clinically recommended behaviours (e.g., physical activity, smoking cessation), participants raised culturally familiar practices, such as natural remedies. Although often raised tentatively for validation, these practices remained meaningful and motivating, offering a manageable starting point that could build momentum for further positive change.

> *'I found out this before what they said in the morning, if you drink warm water with a little bit of lemon in it… it makes your muscles cleaner, bigger, and then, you know, you wash it all down and then makes your muscles clean…. So last week I started doing the warm water with lemon in it, and I feel a lot better… Then you're thinking, oh, you did this in the morning. You don't want to ruin yourself..'* Hafsa, 65 years.

For some, health behaviours were framed as acts of gratitude or spiritual respect.

> *'She feels like our body is actually a gift from God, and we need to look after it, and she is being grateful by looking after it.'* Farah via interpreter, 47 years.

Aligning health messaging with these values can make behaviour change feel personally meaningful and rooted in religious and cultural identity. However, participants' sense of agency could be undermined if their efforts were overlooked. Some expressed disappointment or confusion when, despite avoiding smoking or alcohol, they still faced elevated CVD risk.

For peer review only - http://bmjopen.bmj.com/site/about/guidelines.xhtml

Creating environments where change feels possible

This theme captures how engagement depended on support feeling safe, inclusive, and accessible, with human connection providing the social encouragement and accountability needed to sustain change.

Safe, inclusive, and accessible environments

Participants described environmental and structural barriers that made behaviour change and accessing support feel out of reach. Concerns around affordability, discomfort, and safety varied but had a shared impact: they undermined confidence, increased the emotional cost of participation, and made healthy choices feel less viable, and at times even harmful.

While many participants valued outdoor activity for enjoyment and wellbeing, safety concerns could negate these benefits.

> *'Where we live, we suffer from racist abuse a lot... So you're very scared of stepping outside. You can't really switch off from 'Is someone behind me?', 'What is that person going to do?' It's not a stress-free walk - it's a stressful walk, but then it doesn't really do any good to the heart.'* Rashida, 63 years.

Affordability was another key barrier. Some participants joined subsidised programmes but stopped once funding ended.

> *'For 3 months [the exercise class] was free. Because they were not giving them any more vouchers, she said they asked for £25 per month. At that time, she couldn't pay, so that's why she stopped.'* Nargis via interpreter, 64 years.

Expectations around appearance, body image, and other attendees also contributed to apprehension. Some participants avoided classes due to perceived dress codes.

> *'That was the reason why I was a bit apprehensive [to attend exercise classes], I thought 'Oh gosh, I'm going to have to go and buy a whole wardrobe of gym clothes.' But when*

For peer review only - http://bmjopen.bmj.com/site/about/guidelines.xhtml

we had our first session and I saw everyone in their normal clothes I thought 'OK, that's alright'.' Rashida, 63 years.

Others were initially worried about standing out as the least fit in the room but felt reassured once they saw a range of abilities among attendees. For some women, embarrassment about their bodies led them to prefer women-only spaces, which were valued for comfort and support.

Digital exclusion also limited access, with online booking systems proving a barrier for those lacking the skills or confidence to use them.

The motivating power of human connection

Participants stressed that support was more motivating when it involved social connection and human presence. Group settings and professional guidance reduced the burden of change, made healthy behaviours feel normal. and energised participants to act.

'Even a little bit of walking, it helps. But... I think what it is, it's a little bit of motivation. If one person's doing it, then people will copy... Because nobody wants to do it on their own. So there should be like groups.' Tahira, 65 years.

Even those favouring digital resources expressed that these often felt too passive without human involvement.

'There needs to be... some type of moderation, for example, if there's a health coach or someone working with the individual... Rather than just having information laid out. We have so many apps... but there's no one there telling me or pushing me that it's useful... then I feel like the person can lose track.' Adeel, 40 years.

Cultural familiarity and representation amplified this effect. Professionals with the same ethnic background made advice feel more relevant and safer.

'It helps more, if it's Asian… the health advice is more better, when you're there there's more attention for you, you're more used to the South Asian people… It's like [a] comfortable environment for you.' Imran, 44 years.

For peer review only - http://bmjopen.bmj.com/site/about/guidelines.xhtml

Some felt that rapport built through digital interactions could motivate them to attend in-person services. However, practical barriers such as work, childcare, and transport mean digital alternatives must also be designed to build a sense of human connection to promote active engagement.

Discussion

Key findings

Trusted, personalised information underpins engagement with health behaviour change, reflecting the need for clear, credible explanations that address misinformation and feel personally relevant. Behaviour change also needs to feel manageable and worthwhile through support that recognises everyday efforts, reframes unhelpful cognitions, offers realistic adaptations, and embraces cultural values. Safe, inclusive, accessible environments supported by human connection are essential, while policy and service-level action are required to address wider structural barriers.

Comparison with prior work

NHSHC advice is often generic and inconsistently followed up[5], leaving many, particularly those from socioeconomically deprived groups, without practical guidance. Whereas earlier NHSHC attendees reported being able to find the generic advice provided during NHSHCs online[22], our findings indicate that the current information landscape is contradictory and confusing. This challenge is intensified for those with limited GP access to clarify guidance, disproportionately affecting South Asian populations since the shift to digital consultations[23]. Support must therefore help people navigate conflicting information by providing trusted, practical guidance that is personalised to their circumstances.

Consistent with prior work showing a preference for small, incremental changes[22], we found that acknowledging existing behaviours and tailoring recommendations matters. Participants wanted everyday activities, such as housework and walking, recognised as meaningful contributions. Overlooking these, or valued commitments like abstaining from smoking or alcohol, risks discouragement and undermining self-efficacy, a key factor in risk communication and behaviour change[24].

For peer review only - http://bmjopen.bmj.com/site/about/guidelines.xhtml

Fatalism has been highlighted as influential in health behaviour change[25]; our study shows how it can combine with regret, rigid rules, and unrealistic targets, to create significant cognitive and emotional strain. All-or-nothing mindsets can trigger guilt that discourages further effort, such as avoiding exercise because benefits beyond weight loss are not recognised. This highlights the need for support to counter unhelpful beliefs and reduce the emotional toll of change by reframing it as a process of learning and adaptation rather than strict adherence to rules. Wider social factors, such as racism, further reduced capacity for self-regulation and compounded these challenges.

Earlier studies also noted that some perspectives within minoritised ethnic groups diverge from the biomedical model of health[26,27]. Our findings support this, and identify ways support can leverage cultural beliefs and ensure acceptability. For example, recognising culturally congruent practices while sensitively explaining other risk factors may reduce feelings of unfairness, preserve self-efficacy, and harness cultural values as a driver for change.

Calls for greater representation of minoritised ethnic groups in NHSHC research have highlighted the need to understand how ethnicity and deprivation intersect to shape access and engagement[28]. We addressed this by recruiting adults from minoritised ethnic backgrounds in high-deprivation areas to explore support needs and preferences. The relevance of our findings is reinforced by evidence from other health contexts, such as Iqbal, et al.[29], who report similar barriers, including limited culturally relevant health advice and safe opportunities for physical activity among pregnant British-Pakistani women. These shared barriers indicate systemic issues, pointing towards the need for both system-level change and tailored support.

Strengths and limitations

A key strength of this study was the recruitment of predominantly Asian/ Asian British women living in deprived communities, a group rarely represented in research despite their heightened risk[30] and significant influence on household health practices such as cooking[31]. Using a digital prototype emulated the process of receiving CVD risk feedback and support in a format similar to the digital NHSHC pilot[32]. Although the risk information was hypothetical, the realistic format helped

For peer review only - http://bmjopen.bmj.com/site/about/guidelines.xhtml

participants articulate useful support strategies for both face-to-face and digital delivery, providing novel insights while acknowledging that findings may not fully reflect all minoritised or socioeconomically deprived communities.

Implications

This study adds to the evidence that preventative strategies must be co-designed to be trusted, culturally relevant, and embedded in everyday contexts[7]. For NHSHCs, this means moving beyond generic advice to provide guidance that is personally relevant, credible, and responsive to the priorities of underserved groups.

The availability of referral pathways remains important[33,34], but engagement also depends on whether services feel safe, inclusive, and practically accessible. Both real concerns (e.g. racism, exclusion) and perceived barriers (e.g. assumptions about gym clothing) influence willingness to engage. Commissioners and providers therefore need to prioritise creating supportive environments that feel welcoming, so NHSHCs can function more effectively as gateways to follow-up support and improved health outcomes[35].

Future strategies should build on these findings as a foundation for co-design and evaluation of behaviour change support for minoritised ethnic groups in deprived areas. Priorities include integrating everyday practices and cultural commitments, addressing unhelpful beliefs about CVD prevention, and improving perceptions of support services while ensuring that follow-up options are safe and inclusive. Alongside policy changes to remove structural barriers such as cost, these approaches are critical to making the NHSHC programme more equitable and effective for underserved groups.

Conclusion

NHSHC behaviour change support for minoritised ethnic adults in deprived areas needs to be trusted, culturally relevant, and accessible, with advice grounded in everyday contexts and values. Embedding these principles can improve engagement, strengthen the programme's role as a gateway to follow-up support, and reduce inequalities in CVD prevention.

For peer review only - http://bmjopen.bmj.com/site/about/guidelines.xhtml

For peer review only

References

1. World Health Organisation. Cardiovascular diseases (CVDs), 2021. Available: https://www.who.int/news-room/fact-sheets/detail/cardiovascular-diseases-(cvds).
2. British Heart Foundation. Early heart disease deaths rise to 14-year high, 2024. Available: https://www.bhf.org.uk/what-we-do/news-from-the-bhf/news-archive/2024/january/early-heart-disease-deaths-rise-to-14-year-high
3. Public Health England. Health matters: preventing cardiovascular disease, 2019. Available: https://www.gov.uk/government/publications/health-matters-preventing-cardiovascular-disease/health-matters-preventing-cardiovascular-disease
4. Usher-Smith JA, Mant J, Martin A, et al. *NHS health check programme rapid evidence synthesis.* University of Cambridge, 2017.
5. Usher-Smith J, A., Harte E, MacLure C, et al. Patient experience of NHS health checks: a systematic review and qualitative synthesis. *BMJ Open*. 2017;7(8):e017169. doi: 10.1136/bmjopen-2017-017169
6. Corlett SA, Krska J. Evaluation of NHS Health Checks provided by community pharmacies. *Journal of Public Health*. 2015;38(4):e516-e23. doi: 10.1093/pubmed/fdv153
7. Grice-Jackson T, Rogers I, Ford E, et al. The pre-implementation phase of a project seeking to deliver a community-based CVD prevention intervention (spices-sussex): a qualitative study exploring views and experience relating to intervention development. *Health Promotion Practice.* 2024;25(6):1009-22. doi: 10.1177/15248399231182139
8. Salway R, Sillero-Rejon C, Forte C, et al. A service evaluation of the uptake and effectiveness of a digital delivery of the NHS health check service. *BMJ Open*. 2024;14(11):e091417. doi: 10.1136/bmjopen-2024-091417
9. Malterud K, Siersma VD, Guassora AD. Sample size in qualitative interview studies: guided by information power. *Qualitative Health Research*. 2016;26(13):1753-60. doi: 10.1177/1049732315617444

For peer review only

For peer review only - http://bmjopen.bmj.com/site/about/guidelines.xhtml

10. University of Manchester. Digital health software and platforms, 2024. Available: https://sites.manchester.ac.uk/digital-health-software-and-platforms/

11. Marvel, 2024. Available: https://marvelapp.com/

12. Witteman HO, Fuhrel-Forbis A, Wijeysundera HC, et al. Animated randomness, avatars, movement, and personalization in risk graphics. *Journal of Medical Internet Research.* 2014;16(3):e80. doi: 10.2196/jmir.2895

13. Cowap L, Riley V, Grogan S, et al. "They are saying it's high, but I think it's quite low": exploring cardiovascular disease risk communication in NHS health checks through video-stimulated recall interviews with patients – a qualitative study. *BMC Primary Care.* 2024;25(1) doi: 10.1186/s12875-024-02357-w

14. Schulberg SD, Ferry AV, Jin K, et al. Cardiovascular risk communication strategies in primary prevention. A systematic review with narrative synthesis. *Journal of Advanced Nursing.* 2022;78(10):3116-40. doi: 10.1111/jan.15327

15. Rogers RW. Cognitive and Physiological Processes in Fear Appeals and Attitude Change: A Revised Theory of Protection Motivation. In: Cacioppo J, Petty R, eds. *Social Psychophysiology*. New York: Guilford.1983.

16. Leventhal H, Benyamini Y, Brownlee S, et al. Illness Representations: Theoretical Foundations. In: Petrie KJ, Weinman JA, eds. *Perceptions of Health and Illness*. Amsterdam: Harwood Academic Press 1997:19-45.

17. Pate A, Jiang B, Huang Y-T, et al. CHARIOT: development and internal validation of a cardiovascular health assessment and risk-based intervention optimisation tool. *medRxiv*. 2025;2025.09.18.25336064. doi:10.1101/2025.09.18.25336064

18. McMillan B, Fox S, Lyons M, et al. Using patient and public involvement to improve the research design and funding application for a project aimed at fostering a more collaborative approach to the NHS Health Check: the CaVIAR project (Better Care Via Improved Access to Records). *Res Involv Engagem.* 2018;4:18. doi: 10.1186/s40900-018-0101-7.

19. Braun V, Clarke V. Reflecting on reflexive thematic analysis. *Qualitative Research in Sport, Exercise and Health.* 2019;11(4):589-97. doi: 10.1080/2159676X.2019.1628806

For peer review only - http://bmjopen.bmj.com/site/about/guidelines.xhtml

20. O'Brien BC, Harris IB, Beckman TJ, Reed DA, Cook DA. Standards for reporting qualitative research: A synthesis of recommendations. *Academic Medicine*. 2014;89(9):1245–51. Available: https://journals.lww.com/academicmedicine/fulltext/2014/09000/Standards_for_Reporting_Qualitative_Research__A.21.aspx

21. O'Brien BC, Harris IB, Beckman TJ, Reed DA, Cook DA. The SRQR reporting checklist. In: Harwood J, Albury C, Beyer J de, Schlüssel M, Collins G, eds. *The EQUATOR network reporting guideline platform.* The UK EQUATOR Centre 2025. Available: https:/resources.equator-network.org/reporting-guidelines/srqr/srqr-checklist.docx

22. McNaughton RJ, Shucksmith J. Reasons for (non)compliance with intervention following identification of 'high-risk' status in the NHS Health Check programme. *Journal of Public Health.* 2015;37(2):218-25. doi: 10.1093/pubmed/fdu066

23. Small N, Masood Y, Stevenson F, et al. Exploring the experiences and preferences of South Asian patients' of primary care in England since COVID-19. *Health Expectations.* 2024;27(1) doi: 10.1111/hex.13982

24. French DP, Cameron E, Benton JS, et al. Can communicating personalised disease risk promote healthy behaviour change? a systematic review of systematic reviews. *Annals of Behavioral Medicine.* 2017;51(5):718. doi: 10.1007/s12160-017-9895-z

25. Honey S, Hill K, Murray J, et al. Patients' responses to the communication of vascular risk in primary care: a qualitative study. *Primary Health Care Research & Development.* 2015;16(1):61-70.

26. Hawking MKD, Timmis A, Wilkins F, et al. Improving cardiovascular disease risk communication in NHS Health Checks: a qualitative study. *BMJ Open.* 2019;9(8):e026058. doi: 10.1136/bmjopen-2018-026058

27. Riley R, Coghill N, Montgomery A, et al. The provision of NHS health checks in a community setting: an ethnographic account. *BMC Health Services Research.* 2015;15(1):546. doi: 10.1186/s12913-015-1209-1

For peer review only - http://bmjopen.bmj.com/site/about/guidelines.xhtml

28. Eberhardt J, Kane L, Portman R, et al. Barriers and facilitators of NHS health checks in socioeconomically deprived communities in the north east of england: a qualitative study with peer researchers. *Health Expectations.* 2025;28(2) doi: 10.1111/hex.70199

29. Iqbal H, West J, McEachan RR, Haith-Cooper M. Identifying the health concerns of pregnant british pakistani women living in deprived areas: a qualitative study. *Maternal and Child Health Journal.* 2024;28(3):489-95.

30. Redwood S, Gill PS. Under-representation of minority ethnic groups in research — call for action. *British Journal of General Practice.* 2013;63(612):342-43. doi: 10.3399/bjgp13x668456

31. Vallianatos H, Raine K. Consuming food and constructing identities among arabic and south asian immigrant women. *Food, Culture & Society.* 2008;11(3):355-73. doi: 10.2752/175174408X347900

32. Forte C, Grey EB, Jessiman P, et al. Exploring service users' and healthcare professionals' experience of digital and face-to-face Health Checks in England: a qualitative study. *BMJ Open.* 2025;15(3):e090492. doi: 10.1136/ bmjopen-2024-090492

33. Alageel S, Gulliford MC, McDermott L, Wright AJ. Implementing multiple health behaviour change interventions for cardiovascular risk reduction in primary care: a qualitative study. *BMC Family Practice.* 2018;19(1):171. doi: 10.1186/s12875-018-0860-0

34. Gadsby EW, Krska J, Duddy C, et al. The NHS Health Check programme: a survey of programme delivery in England before and after the Covid-19 pandemic response. *NIHR Open Research.* 2023;3:32. doi: 10.3310/nihropenres.13436.2

35. Duddy C, Gadsby E, Hibberd V, et al. What happens after an NHS Health Check? A survey and realist review. *Health and Social Care Delivery Research.* 2022;11(12). doi: 10.3310/RGTH4127

For peer review only - http://bmjopen.bmj.com/site/about/guidelines.xhtml

Declarations

Acknowledgements

We thank the staff of the community organisation for their assistance with recruitment and translation, and the community members who participated for their invaluable contributions to this research. We also thank Simon Foster for developing the digital prototype used in the study. We extend our gratitude to the PPI members for their important input.

Contributors

SG led the study design and data collection, coded and analysed the data, and drafted the manuscript. YKB, DPF, and BM contributed to the conceptualisation, supervised coding and analysis, and reviewed the manuscript. All authors read and approved the final manuscript.

Funding

This study was funded by a PhD studentship funded by the EMIS National User Group (https://emisnug.org.uk/). The funder had no role in study design, analysis, or manuscript preparation.

Competing interests

S.G. was awarded a PhD Scholarship (£94,498; 01/10/2023 – 30/09/2026) to develop an interactive patient-facing cardiovascular risk calculator; the funding was originally granted to B.M., D.P.F., and Y.K.B.

D.F. is a National Institute for Health and Care Research (NIHR) Senior Investigator (NIHR305827).

B.M. is in receipt of funding from the National Institute for Health Research School for Primary Care Research (NIHR SPCR) and Endeavour Health Charitable Trust to develop a Cardiovascular Health Assessment and Risk-based Intervention Optimisation Tool (CHARIOT), a patient-facing cardiovascular risk tool aimed at demonstrating potential risk reductions associated with behaviours such as smoking cessation or weight loss (https://www.spcr.nihr.ac.uk/research/projects/chariot-a-cardiovascular-health-assessment-and-risk-based-intervention-optimisation-tool-embedded-within-the-patient-facing-health-record). B.M.'s support from the NIHR (£55,000; 01/07/2023 – 31/12/2024)

For peer review only

For peer review only - http://bmjopen.bmj.com/site/about/guidelines.xhtml

for CHARIOT was used to pay for CPRD and Evergreen Life data licenses and patient and public involvement; and from the Endeavour Health Charitable Trust (£81,662; 01/07/2023 – 31/12/2024), was used to employ a Research Fellow (Alexander Pate) to develop the CHARIOT algorithm.

Patient and public involvement

Patients and/or the public were involved in the design of this research. See the Methods section for further details.

Ethics approval

This study received University of Manchester Research Ethics approval (REF 2024-19916-37529). All participants provided written or electronic informed consent.

Data availability statement

The data from this study are anonymised but contain context-specific narratives that could risk participant identification; to maintain confidentiality, they are not publicly available and cannot be shared.

For peer review only

For peer review only - http://bmjopen.bmj.com/site/about/guidelines.xhtml

Figure 1. Screenshots of the digital prototype presenting CVD risk information and signposting to behaviour change support.

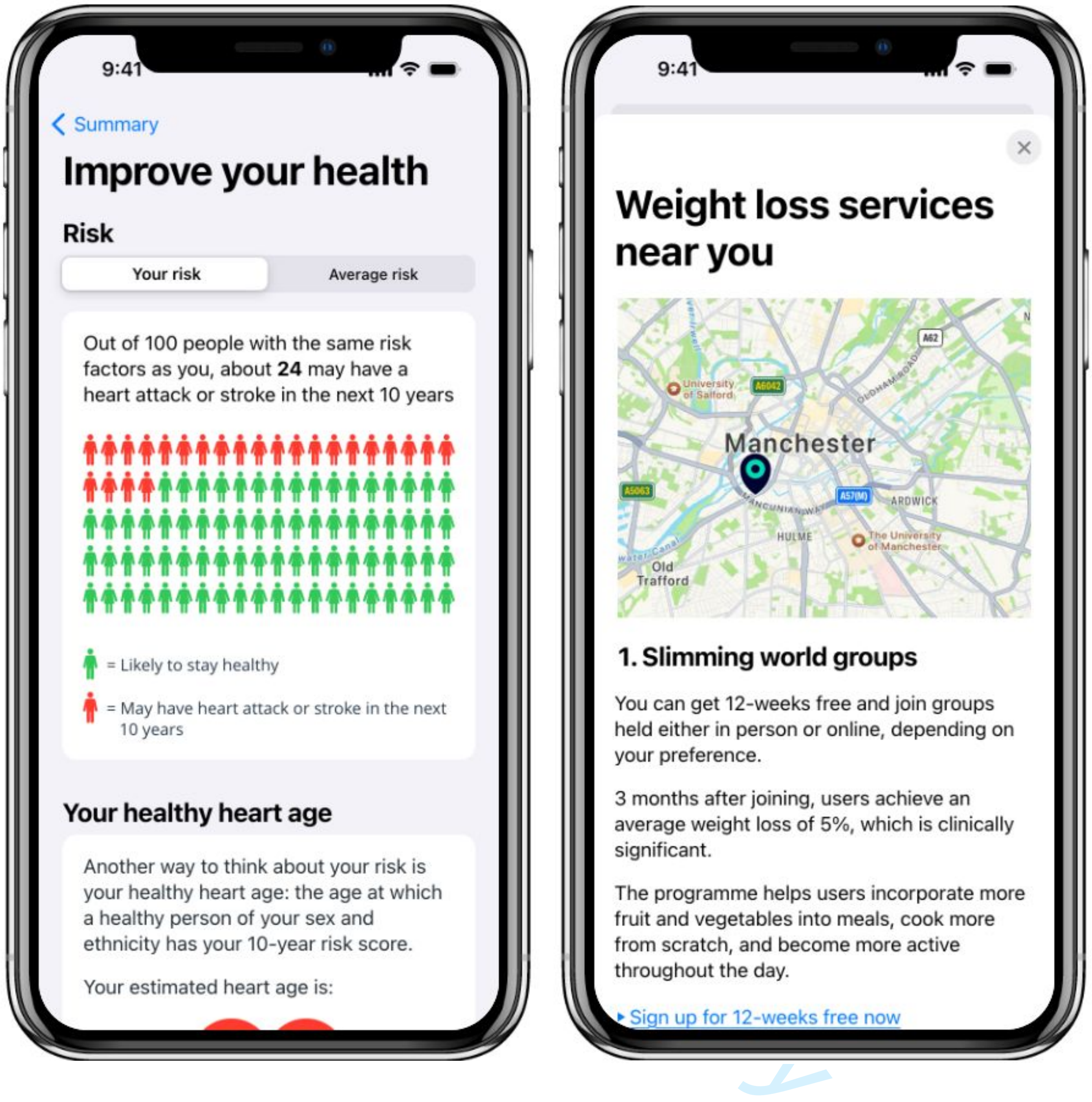

Supplementary File

Supplement A

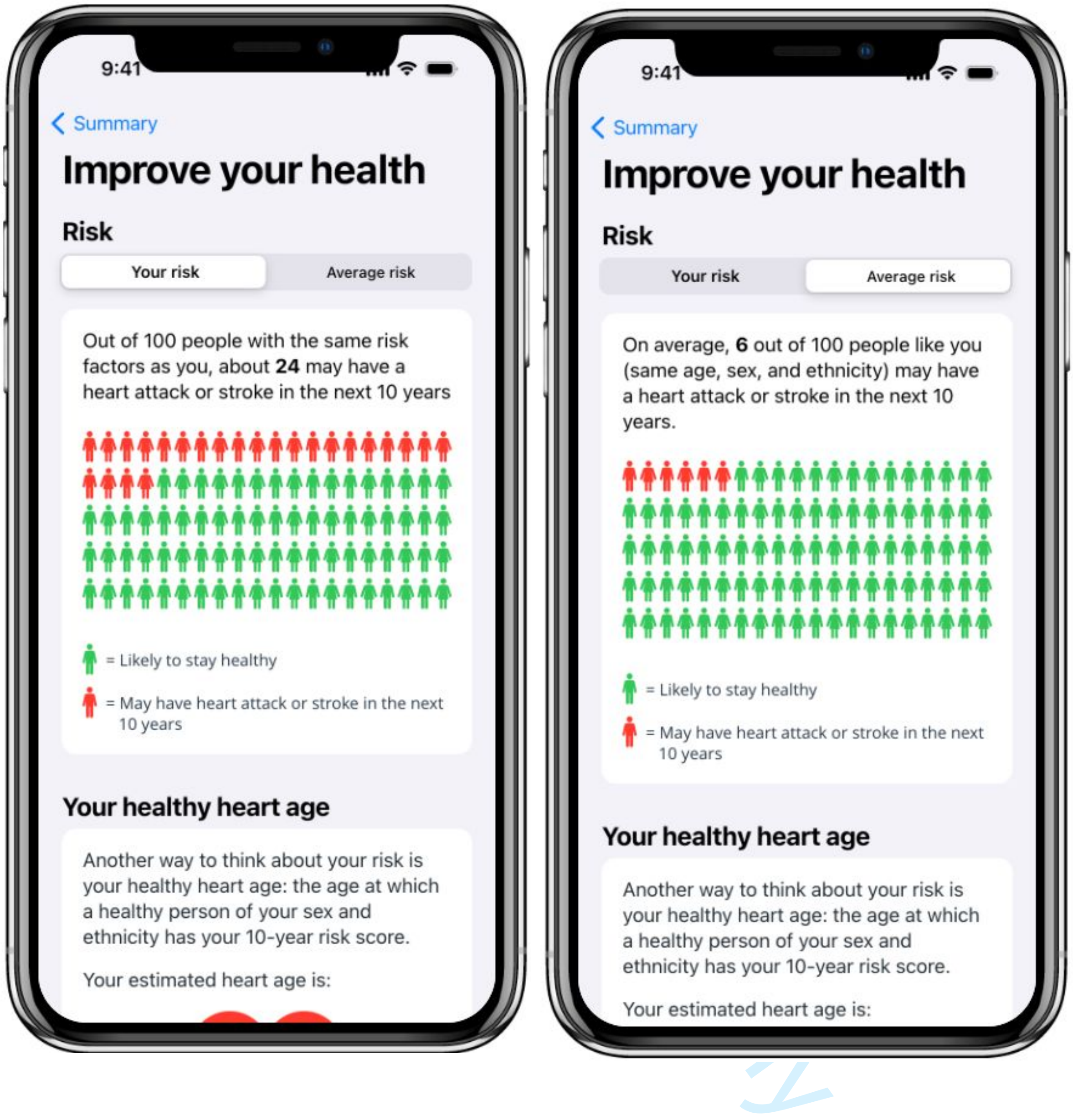

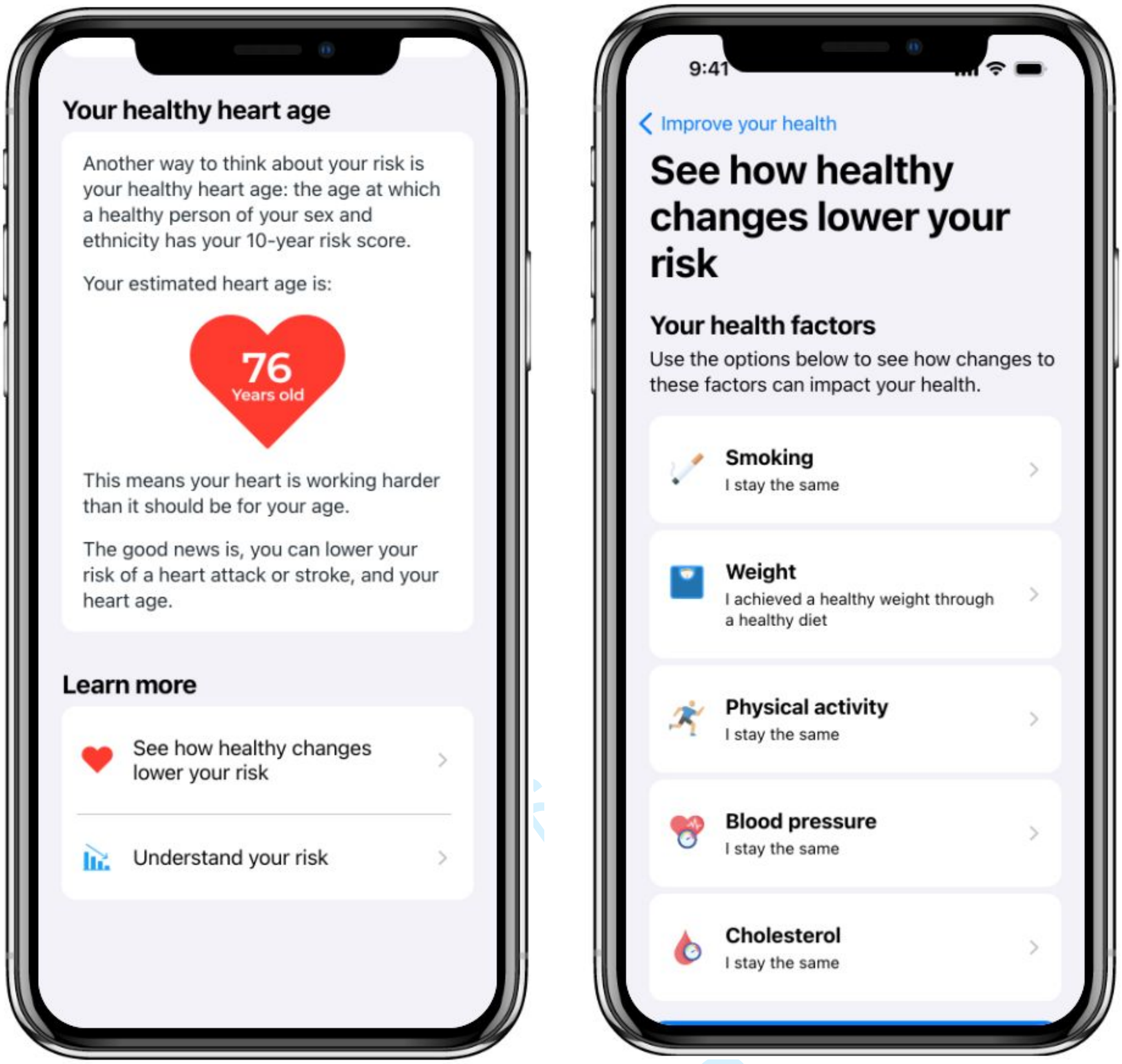
Your healthy heart age
Another way to think about your risk is your healthy heart age: the age at which a healthy person of your sex and ethnicity has your 10-year risk score.
Your estimated heart age is:
76
Years old
This means your heart is working harder than it should be for your age.
The good news is, you can lower your risk of a heart attack or stroke, and your heart age.
Learn more
See how healthy changes lower your risk
Understand your risk
9:41
Improve your health
See how healthy changes lower your risk
Your health factors
Use the options below to see how changes to these factors can impact your health.
Smoking
I stay the same
Weight
I achieved a healthy weight through a healthy diet
Physical activity
I stay the same
Blood pressure
I stay the same
Cholesterol
I stay the same

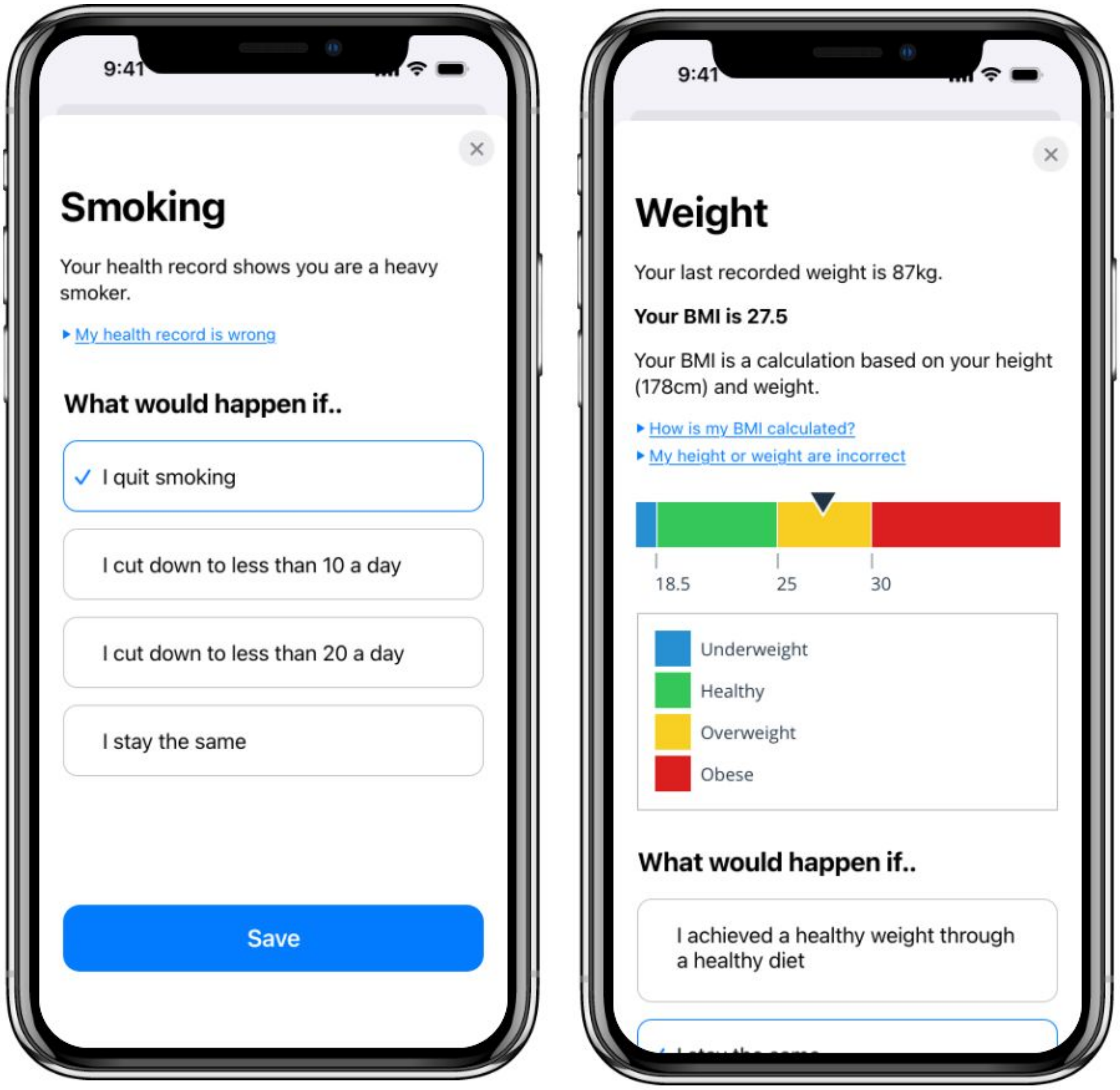
9:41
Smoking
Your health record shows you are a heavy smoker.
My health record is wrong
What would happen if..
I quit smoking
I cut down to less than 10 a day
I cut down to less than 20 a day
I stay the same
Save
9:41
Weight
Your last recorded weight is 87kg.
Your BMI is 27.5
Your BMI is a calculation based on your height (178cm) and weight.
How is my BMI calculated?
My height or weight are incorrect
18.5
25
30
Underweight
Healthy
Overweight
Obese
What would happen if..
I achieved a healthy weight through a healthy diet

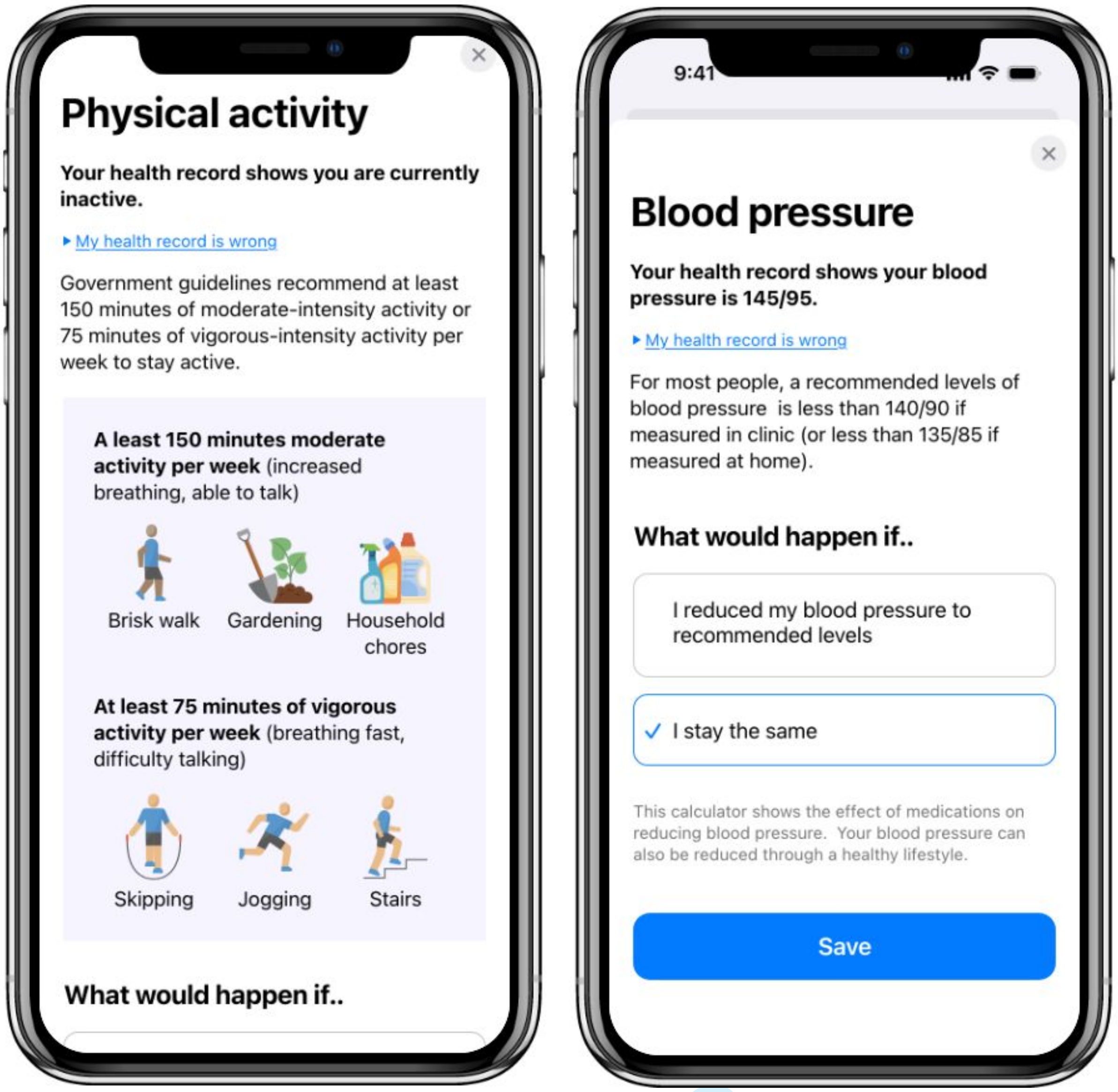
Physical activity
Your health record shows you are currently inactive.
My health record is wrong
Government guidelines recommend at least 150 minutes of moderate-intensity activity or 75 minutes of vigorous-intensity activity per week to stay active.
A least 150 minutes moderate activity per week (increased breathing, able to talk)
Brisk walk
Gardening
Household chores
At least 75 minutes of vigorous activity per week (breathing fast, difficulty talking)
Skipping
Jogging
Stairs
What would happen if..
9:41
Blood pressure
Your health record shows your blood pressure is 145/95.
My health record is wrong
For most people, a recommended levels of blood pressure is less than 140/90 if measured in clinic (or less than 135/85 if measured at home).
What would happen if..
I reduced my blood pressure to recommended levels
I stay the same
This calculator shows the effect of medications on reducing blood pressure. Your blood pressure can also be reduced through a healthy lifestyle.
Save

only

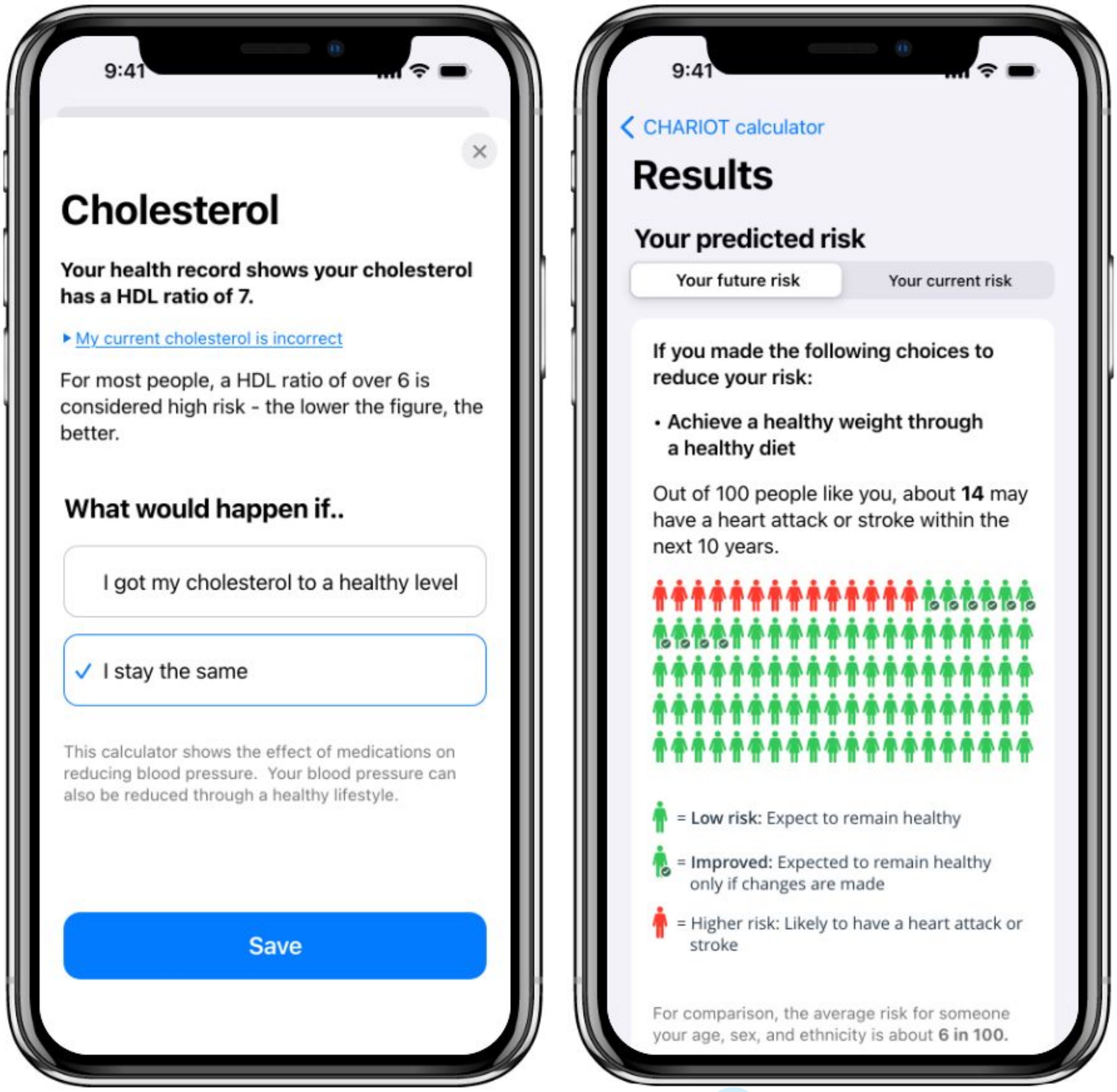
9:41
Cholesterol
Your health record shows your cholesterol has a HDL ratio of 7.
My current cholesterol is incorrect
For most people, a HDL ratio of over 6 is considered high risk - the lower the figure, the better.
What would happen if..
I got my cholesterol to a healthy level
I stay the same
This calculator shows the effect of medications on reducing blood pressure. Your blood pressure can also be reduced through a healthy lifestyle.
Save
9:41
CHARIOT calculator
Results
Your predicted risk
Your future risk
Your current risk
If you made the following choices to reduce your risk:
• Achieve a healthy weight through a healthy diet
Out of 100 people like you, about 14 may have a heart attack or stroke within the next 10 years.
= Low risk: Expect to remain healthy
= Improved: Expected to remain healthy only if changes are made
= Higher risk: Likely to have a heart attack or stroke
For comparison, the average risk for someone your age, sex, and ethnicity is about 6 in 100.

only

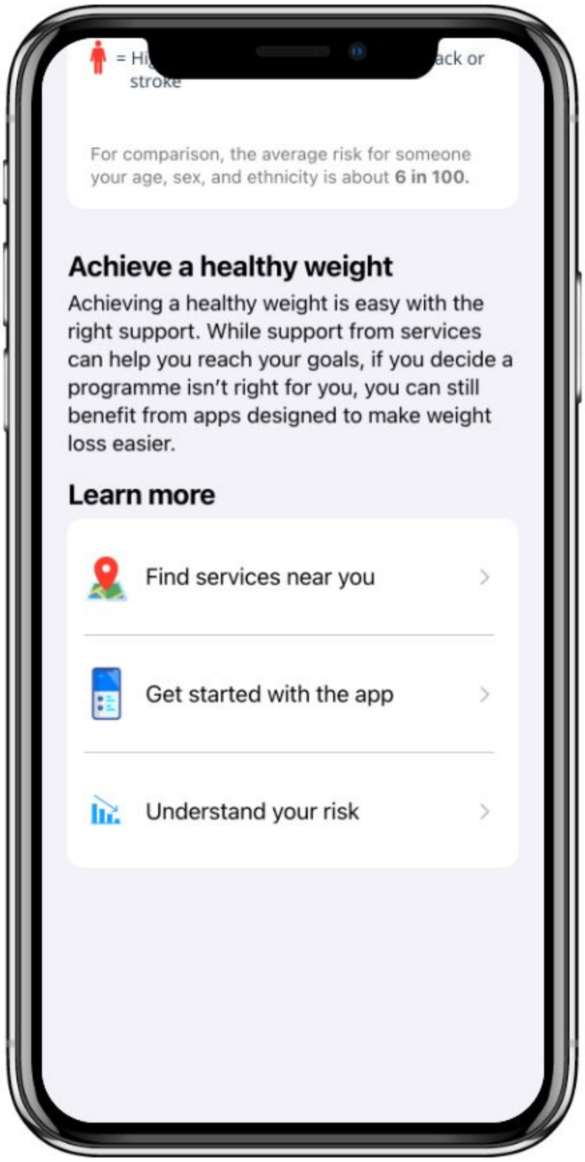
For comparison, the average risk for someone your age, sex, and ethnicity is about 6 in 100.
Achieve a healthy weight
Achieving a healthy weight is easy with the right support. While support from services can help you reach your goals, if you decide a programme isn't right for you, you can still benefit from apps designed to make weight loss easier.
Learn more
Find services near you
Get started with the app
Understand your risk

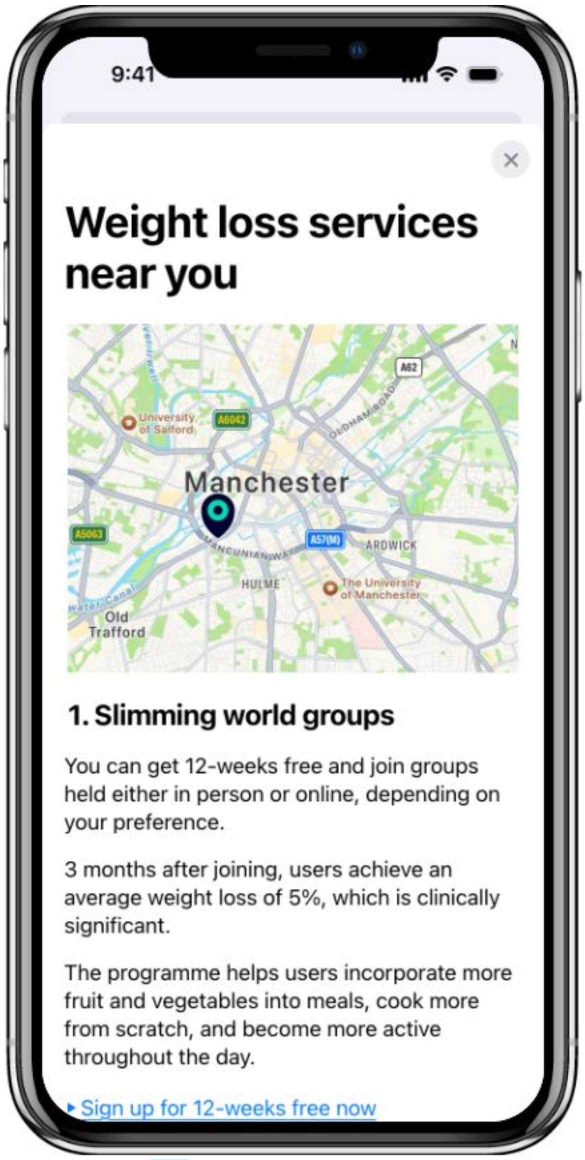
9:41
Weight loss services near you
Manchester
University of Salford
A6042
A62
A5063
A57(M)
ARDWICK
HULME
The University of Manchester
Old Trafford
1. Slimming world groups
You can get 12-weeks free and join groups held either in person or online, depending on your preference.
3 months after joining, users achieve an average weight loss of 5%, which is clinically significant.
The programme helps users incorporate more fruit and vegetables into meals, cook more from scratch, and become more active throughout the day.
Sign up for 12-weeks free now

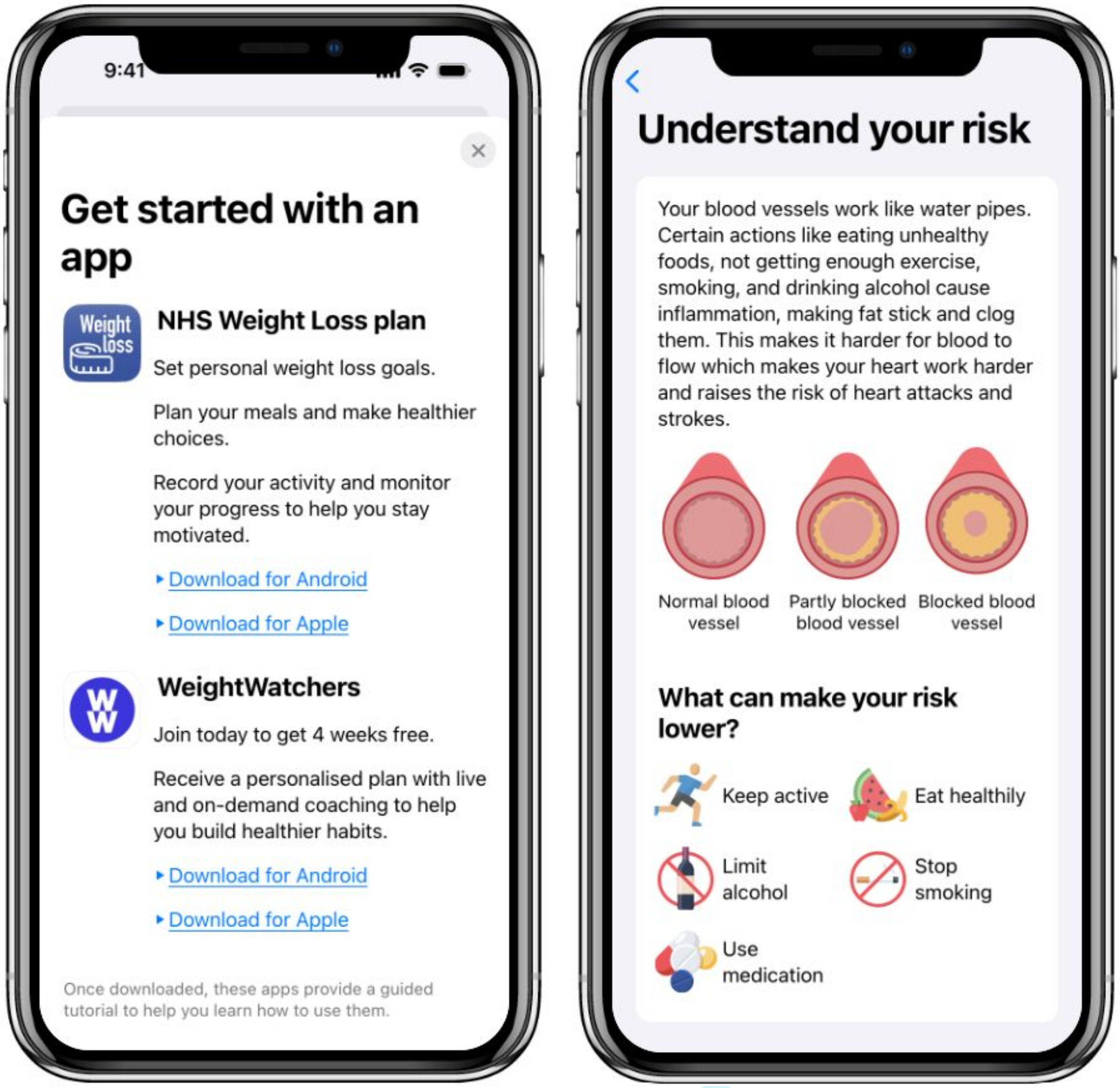
9:41
Get started with an app
Weight loss
NHS Weight Loss plan
Set personal weight loss goals.
Plan your meals and make healthier choices.
Record your activity and monitor your progress to help you stay motivated.
Download for Android
Download for Apple
WeightWatchers
Join today to get 4 weeks free.
Receive a personalised plan with live and on-demand coaching to help you build healthier habits.
Download for Android
Download for Apple
Once downloaded, these apps provide a guided tutorial to help you learn how to use them.
Understand your risk
Your blood vessels work like water pipes. Certain actions like eating unhealthy foods, not getting enough exercise, smoking, and drinking alcohol cause inflammation, making fat stick and clog them. This makes it harder for blood to flow which makes your heart work harder and raises the risk of heart attacks and strokes.
Normal blood vessel
Partly blocked blood vessel
Blocked blood vessel
What can make your risk lower?
Keep active
Eat healthily
Limit alcohol
Stop smoking
Use medication

only

The SRQR reporting checklist

For checking that qualitative health research articles can be understood and used by everyone

> **Note**
>
> If you have not used a reporting guideline before, read about how and why to use them and check whether SRQR is the most applicable reporting guideline for your work.
>
> Reporting guidelines are most useful when used early in research. When writing a manuscript or application, consider using the Full Guidance where you'll see explanations and examples for each item.
>
> After writing, demonstrate adherence by completing this checklist:
>
> 1. Specify where each item is described (see Note 1).
> 2. Cite this checklist (See Note 2).
> 3. Include your completed checklist as a supplement when submitting to a journal so that future readers can use it to find information.

	Item Description	Location (or reason for not reporting)
**Title & Abstract**		
Title	Describe the nature and topic of the study. Identify the study as qualitative or indicate the approach or data collection methods.	Title page
Abstract	Summarise the key elements of the study using the abstract format of the intended publication.	Title page
**Introduction**		
Problem Formulation	Describe the problem/phenomenon studied, its significance, relevant theory and empirical work, and gaps in current knowledge.	Introduction; paragraphs 1, 2, 3, 4
Purpose or research question	Describe the purpose of the study and specific objectives or questions.	Introduction; paragraph 4. Aim
**Methods**		
Qualitative approach and research paradigm	Describe your qualitative approach, your guiding theory (if appropriate), and research paradigm, and reasons for your choices.	Methods, Data collection; paragraphs 1 & 2
Researcher characteristics and reflexivity	Describe how researchers' characteristics may influence the research, including personal attributes, qualifications/experience, relationship with	Methods, Analysis; paragraph 2

For peer review only

	participants, assumptions, and/or presuppositions; potential or actual interaction between researchers' characteristics and the research questions, approach, methods, results and/or transferability.	
Context	Describe the setting/site(s) in which the study was conducted, why it was selected, and any other salient contextual factors that may influence the study.	Methods, Participant recruitment; paragraph 1
Sampling strategy	Describe how and why research participants, documents, or events were selected; criteria for deciding when no further sampling was necessary, and the rationale for those criteria.	Methods, Participant recruitment; paragraph 1 & 2
Ethical issues pertaining to human subjects	Describe any approval by an appropriate ethics review board and participant consent, or explain any lack thereof. Describe any other confidentiality and data security issues.	Methods, Study design
Data collection methods	Describe the types of data collected; details of data collection procedures including (as appropriate) start and stop dates of data collection and analysis, iterative process, triangulation of sources/methods, and modification of procedures in response to evolving study findings. Describe your rationale for these choices.	Methods, Data collection
Data collection instruments and technologies	Describe any instruments (e.g., interview guides, questionnaires) and devices (e.g., audio recorders) used for data collection; describe if/how the instrument(s) changed over the course of the study.	Methods, Materials
Units of study	Describe the number and relevant characteristics of participants, documents, or events included in the study. Describe the level of participation.	Results; paragraph 1
Data processing	Describe the methods for processing data prior to and during analysis, including transcription, data entry, data management and security, verification of data integrity, data coding, and anonymisation / deidentification of excerpts.	Methods, Analysis; paragraph 1
Data analysis	Describe the process by which inferences, themes, etc. were identified and developed, including the researchers involved in data analysis; usually references a specific paradigm or approach. Describe why you chose this process.	Methods, Analysis; paragraph 1
Techniques to enhance trustworthiness	Describe any techniques to enhance trustworthiness and credibility of data analysis,(e.g., member checking, triangulation, audit trail). Describe why you chose these techniques.	Methods, Analysis; paragraph 2

**Results**		
Synthesis and interpretation	Describe the main findings (e.g., interpretations, inferences, and themes); might include development of a theory or model, or integration with prior research or theory.	Results; Table 1. & First paragraph beneath each theme heading.
Links to empirical data	Provide evidence (e.g., quotes, field notes, text excerpts, photographs) to substantiate analytic findings.	Results; embedded throughout (e.g., paragraph 4).
**Discussion**		
Integration with prior work, implications, transferability, and contribution(s) to the field	Summarize the main findings, explain how findings and conclusions connect to, support, elaborate on, or challenge conclusions of earlier scholarship; discuss the scope of application/generalizability; identify unique contribution(s) to scholarship in a discipline or field.	Discussion; paragraph 1-9
Limitations	Discuss the trustworthiness and limitations of findings	Discussion; paragraph 7
**Other**		
Conflicts of interest	Describe any potential sources of influence or perceived influence on study conduct and conclusions. Describe how these were managed.	Declarations, Competing interests
Funding	Describe sources of funding and other support. Describe the role of funders in data collection, interpretation, and reporting.	Declarations, Funding

1 How to specify where content is

Tell the reader where they can find information. E.g.,

- Results; paragraph 2
- Methods, Participants; paragraphs 1 & 2.
- Table 3
- Supplement B, para. 4

If you have chosen not to describe an item, explain why. You can do this in the checklist, or as a note below it.

You can describe items in the article body, or in tables, figures, or supplementary materials, and should prioritize items you feel are most important to your intended audience. The order of items in your manuscript does not need to match the order of items in this checklist. You can decide how best to structure your work.

2 How to cite

Describe how you used SRQR at the end of your Methods section, referencing the resources you used e.g.,

For peer review only

‘We used the SRQR reporting guideline(1) to draft this manuscript, and the SRQR reporting checklist(2) when editing, included in supplement A’

If you use a reporting checklist, remember to include it as a supplement when publishing so that readers can easily find information and see how you have interpreted the guidance.

1. O’Brien BC, Harris IB, Beckman TJ, Reed DA, Cook DA. Standards for reporting qualitative research: A synthesis of recommendations. Academic Medicine [Internet]. 2014 Sep;89(9):1245–51. Available from: https://journals.lww.com/academicmedicine/fulltext/2014/09000/Standards_for_Reporting_Qualitative_Research__A.21.aspx

2. O’Brien BC, Harris IB, Beckman TJ, Reed DA, Cook DA. The SRQR reporting checklist. In: Harwood J, Albury C, Beyer J de, Schlüssel M, Collins G, editors. The EQUATOR network reporting guideline platform [Internet]. The UK EQUATOR Centre; 2025. Available from: https:/resources.equator-network.org/reporting-guidelines/srqr/srqr-checklist.docx

For peer review only

Understanding Preferences for Behaviour Change Support as part of the NHS Health Check: A Qualitative Study with Adults from Underserved Minoritised Ethnic Communities

Journal:	*BMJ Open*
Manuscript ID	bmjopen-2025-111413.R1
Article Type:	Original research
Date Submitted by the Author:	15-Dec-2025
Complete List of Authors:	Griffiths, Sophie; The University of Manchester School of Health Sciences, Centre for Health Psychology<br>Bartlett, Yvonne Kiera; The University of Manchester, The Division of Psychology and Mental Health<br>French, David; The University of Manchester School of Health Sciences, Centre for Health Psychology<br>McMillan, Brian; The University of Manchester School of Health Sciences, Centre for Primary Care and Health Services Research
**Primary Subject Heading**:	Qualitative research
Secondary Subject Heading:	Public health, Health services research
Keywords:	Health Services, PUBLIC HEALTH, QUALITATIVE RESEARCH, Preventive Health Services, Patient Preference, Health Equity

I, the Submitting Author has the right to grant and does grant on behalf of all authors of the Work (as defined in the below author licence), an exclusive licence and/or a non-exclusive licence for contributions from authors who are: i) UK Crown employees; ii) where BMJ has agreed a CC-BY licence shall apply, and/or iii) in accordance with the terms applicable for US Federal Government officers or employees acting as part of their official duties; on a worldwide, perpetual, irrevocable, royalty-free basis to BMJ Publishing Group Ltd ("BMJ") its licensees and where the relevant Journal is co-owned by BMJ to the co-owners of the Journal, to publish the Work in this journal and any other BMJ products and to exploit all rights, as set out in our licence.

The Submitting Author accepts and understands that any supply made under these terms is made by BMJ to the Submitting Author unless you are acting as an employee on behalf of your employer or a postgraduate student of an affiliated institution which is paying any applicable article publishing charge ("APC") for Open Access articles. Where the Submitting Author wishes to make the Work available on an Open Access basis (and intends to pay the relevant APC), the terms of reuse of such Open Access shall be governed by a Creative Commons licence – details of these licences and which Creative Commons licence will apply to this Work are set out in our licence referred to above.

Other than as permitted in any relevant BMJ Author's Self Archiving Policies, I confirm this Work has not been accepted for publication elsewhere, is not being considered for publication elsewhere and does not duplicate material already published. I confirm all authors consent to publication of this Work and authorise the granting of this licence.

For peer review only

Understanding Preferences for Behaviour Change Support as part of the NHS Health Check: A Qualitative Study with Adults from Underserved Minoritised Ethnic Communities

Authors

Sophie Griffiths[1]*, Dr Y Kiera Bartlett[1], Prof David P French[1], Dr Brian McMillan[2]

*Corresponding author: Sophie Griffiths, Coupland Building 1, Manchester, M15 6FH.

sophie.griffiths@manchester.ac.uk, 0161 306 9078

Affiliations

[1]Centre for Health Psychology, Manchester, England, [2]Centre for Primary Care and Health Services Research, Manchester, England.

Email addresses

Sophie Griffiths sophie.griffiths@manchester.ac.uk

Y Kiera Bartlett kiera.bartlett@manchester.ac.uk

David P French david.french@manchester.ac.uk

Brian McMillan brian.mcmillan@manchester.ac.uk

Keywords

Health Behavior; Preventive Health Services; Health Services Needs and Demand; Ethnic Groups; Socioeconomic Factors

Word count

3969/ 4000

For peer review only - http://bmjopen.bmj.com/site/about/guidelines.xhtml

For peer review only

Abstract (Word count: 300/ 300)

Introduction: NHS Health Checks provide individuals with cardiovascular disease risk scores alongside advice and signposting to behaviour change support. A particular problem is that the support people receive is often poorly delivered, absent, or not tailored to the needs of people in deprived communities, which risks exacerbating health inequalities. Improving this support is critical if NHS Health Checks are to achieve their goals of prevention and equity.

Objectives: To explore needs for and preferences in behaviour change support among adults in deprived areas, using a digital prototype presenting cardiovascular disease risk information and signposting to services.

Design: A longitudinal qualitative study involving focus groups and semi-structured follow-up interviews.

Participants and setting: Adults from minoritised ethnic groups eligible for NHS Health Checks, recruited online and through a community centre, with both methods targeting high-deprivation areas.

Method: Participants were first shown the digital prototype in focus groups to generate discussion. Follow-up interviews captured more in-depth reflections on needs for behaviour change support. Data were analysed using reflexive thematic analysis.

Results: We conducted four focus groups and 20 follow-up interviews with 23 adults, predominantly of South Asian ethnicity living in areas of high deprivation. We developed three themes: (1) Trusted information to counter confusion and misinformation; (2) Support that makes change feel possible and meaningful, through culturally and personally relevant advice that addresses unhelpful beliefs about risk reduction and behaviour change; and (3) Ensuring access to inclusive, socially connected environments that feel supportive and conducive to action.

Conclusions: For minoritised ethnic adults in deprived areas, NHS Health Check support should build on everyday practices and foster positive perceptions of services. Alongside service-level changes, policy action is needed to remove structural barriers (e.g., cost, safety) that limit people's

For peer review only - http://bmjopen.bmj.com/site/about/guidelines.xhtml

ability to act on advice. Such changes could enhance the programme's contribution to reducing inequalities in cardiovascular disease prevention.

Strengths and limitations of this study

- This study addresses a key gap in NHS Health Check literature by recruiting predominantly Asian/Asian British women from deprived communities, a group underrepresented despite heightened risk.
- A digital prototype presenting CVD risk in a realistic NHS Health Check format was used to explore support preferences, even though the risk information was hypothetical.
- Focus groups facilitated discussion of participants' support needs, with follow-up interviews providing further reflection and depth.

For peer review only

For peer review only - http://bmjopen.bmj.com/site/about/guidelines.xhtml

Introduction

Cardiovascular disease (CVD) is the leading cause of death worldwide, responsible for nearly one-third of all annual deaths[1]. In the UK, over 7.6 million people live with CVD[2]. Modifiable conditions such as high cholesterol, high blood pressure, and type 2 diabetes increase CVD risk and are strongly influenced by social and economic determinants including deprivation[3]. Effective prevention therefore requires engaging people whose health behaviours are shaped by intersecting social and economic pressures.

The NHS Health Check (NHSHC) invites adults aged 40–74 years without established CVD to assess and manage their risk. Uptake is lowest in communities with the greatest burden: socioeconomically deprived neighbourhoods and minoritised ethnic groups[4]. To improve reach, commissioners have extended delivery beyond general practice into community venues such as pharmacies, mosques, and pop-up clinics, which tend to attract higher-risk and underserved groups. These venues facilitate NHSHC attendance because such community assets mobilise social capital, including informal networks, shared norms, and trust[5].

Attendance alone, however, does not guarantee improved cardiovascular outcomes. Benefits depend on whether people understand and act on the advice received[6]. While policy and research have focused on increasing uptake, less attention has been given to the equally critical step of supporting behaviour change. The generic advice often provided in NHSHCs[7] may be particularly ineffective for those facing combined socioeconomic and ethnic disadvantage. Follow-up support also appears under-utilised: a pharmacy-based study found low uptake of recommended follow-up, representing a missed opportunity to assist those most in need[6].

Prior qualitative research in a socioeconomically deprived area in south England concluded that CVD prevention support should be co-designed with local organisations, delivered by trusted community members, and centred on peer bonds and enjoyment rather than generic health messages[8]. However, that study explored general preventative preferences rather than needs formed after receiving CVD risk information and did not consider how ethnicity and deprivation intersect to compound

For peer review only - http://bmjopen.bmj.com/site/about/guidelines.xhtml

disadvantage. To address these gaps, we showed adults from minoritised ethnic and socioeconomically deprived backgrounds a digital prototype presenting hypothetical CVD risk information and support options, similar to the format used in the digital NHSHC pilot[9], providing a realistic prompt for discussing their support needs.

Aim

To identify needs and preferences for behaviour change support among adults from socioeconomically deprived and minoritised ethnic backgrounds, using a digital prototype to ground discussions in the context of NHSHCs.

Method

Study design

This longitudinal qualitative study used focus groups and follow-up one-to-one semi-structured interviews. The study received University of Manchester Research Ethics approval (2024-19916-37529). All participants provided written or electronic consent.

Participant recruitment

Participants were recruited through in-person and online approaches targeting socioeconomically deprived areas. Deprivation was assessed using the English Index of Multiple Deprivation (IMD), which ranks individual's postcodes in deciles range from 1 (most deprived) to 10 (least deprived) based on multiple factors including income and employment. The community centre used for in-person recruitment was in Greater Manchester, in an area ranked in the most deprived IMD decile (1) and primarily served South Asian communities. In-person recruitment was facilitated by community centre staff, who invited eligible participants from their existing networks. Additional recruitment took place via social media platforms including local community groups and classified ad sites, focusing on high-deprivation areas of Greater Manchester.

Eligibility criteria were the same as those for the NHSHC, namely adults aged 40 to 74 years old with no previous diagnosis of CVD. Individuals outside this age range or with a prior diagnosis of CVD

For peer review only - http://bmjopen.bmj.com/site/about/guidelines.xhtml

were therefore excluded. We judged the final sample of participants to have sufficient information power[10] based on the study's defined aim, the quality of dialogue, and the alignment of participant characteristics with the research focus on individuals from minoritised ethnic groups living in deprived areas.

No participant was required to interact directly with the digital prototype, which was demonstrated by the researcher via screen share during in-person or virtual sessions. This approach, combined with recruitment through a community centre, reduced potential bias toward participants already comfortable with technology.

Data collection

Focus groups explored initial responses to the digital prototype, beginning in October 2024 and ending in April 2025. Participants were told that in practice, risk information would be linked to electronic health records. Discussions covered understanding of CVD risk, attitudes towards behaviour change, perceptions of signposting, and preferred support types. A short demographic questionnaire was completed at the end of each group.

Participants were invited to a one-to-one follow-up interview at least one week after the focus group to reflect on the prototype and discuss personal needs and preferences in greater depth.

All focus groups and interviews were conducted by the first author (SG) who has expertise in qualitative methods and behaviour change. Sessions were held via Zoom or at the community centre, with an interpreter available for Urdu-English translation as needed. The interpreter was briefed on the study aims, confidentiality, and the importance of verbatim translation. SG spoke directly to the participant, and the interpreter translated in the third person. The prototype was iterated between each focus group in line with participant feedback.

Participants received a £30 retail voucher for the focus group and an additional £15 voucher for the follow-up interview as a thank you.

For peer review only - http://bmjopen.bmj.com/site/about/guidelines.xhtml

Materials

We used questionnaires (Qualtrics XM or paper) to determine participant eligibility and collect the following demographic information: age (years), gender, and ethnicity (2011 England and Wales Census categories), highest education qualification, and full postcode.

The digital prototype of an app was developed by a specialist Digital Health Software Team based at the University of Manchester[11] using Marvel software[12]. The first iteration was informed by patient and public involvement (PPI), literature on risk communication[13-15], and psychological theory[16,17]. Each iteration was refined through a separate co-design process, with details described in a related study. It included visual CVD risk displays alongside a simulated CVD risk estimation tool, illustrating potential risk reductions from health behaviour changes based on a cardiovascular risk prediction algorithm currently under development[18], as well as brief cardiovascular health education (including national physical activity guidelines) and referral/ signposting to weight management services and digital tools (see Figure 1 and Supplementary Material S1).

Figure 1. Screenshots of the digital prototype presenting CVD risk information and signposting to behaviour change support.

A focus group schedule guided participants through the prototype and facilitated discussions specific to its design and preferences for support after receiving CVD information, which were explored in more depth in interviews. Both focus group and interview topic guides were developed based on previous literature to comprehensively address the research aims and were iteratively refined following feedback from an initial subset of participants to ensure clarity and relevance. The topic guides are provided in Supplementary Material S2.

Patient and public involvement

We involved PPI members from the development stage to help shape the study focus and priorities of this research[19]. All participant-facing documents were reviewed to enhance accessibility. In July 2024, an online session with three PPI members informed the first prototype iteration.

For peer review only - http://bmjopen.bmj.com/site/about/guidelines.xhtml

Analysis

Focus groups and interviews were audio-recorded, transcribed verbatim (including contributions mediated by the interpreter), and anonymised. Transcripts were analysed using reflexive thematic analysis[20]. Initial coding by SG focused on semantic codes, capturing explicit statements, while later stages of analysis became more interpretative, using latent codes to explore underlying meanings and contextual influences on participants' accounts.

Reflexivity was maintained through reflexive notes, an audit trail documenting analytic decisions, and regular discussions of interpretations of the data with co-authors[20]. In line with this reflexive approach, SG considered how her own identity as a white woman may have shaped interactions with participants - most of whom were also women. This shared gender identity may have built rapport and encouraged participants to share more openly. While SG's outsider perspective in relation to cultural background may have limited the full appreciation of cultural nuances, it also encouraged a more open approach to clarifying participants' perspectives and avoiding assumptions. This outsider status may have prompted participants to elaborate more fully, resulting in richer data. Member-checking was not undertaken, as verification-oriented approaches are conceptually inconsistent with reflexive thematic analysis[21]. Credibility was instead ensured through transparent documentation and reflexive, collaborative analytic processes consistent with the epistemological foundations of reflexive thematic analysis.

This manuscript was prepared in line with the Standards for Reporting Qualitative Research (SRQR) guideline[22] and checklist[23], provided in Supplementary Material S3.

Results

A total of 23 participants attended four focus groups: with 20 completing follow-up interviews. Six were recruited online and 17 via a community centre. Many participants held educational qualifications which did not directly align with UK qualification frameworks (e.g. from Pakistan). Given the range of educational systems these data were not categorised further. An interpreter assisted with translation for six participants. Sample characteristics are summarised in Table 1.

For peer review only

For peer review only - http://bmjopen.bmj.com/site/about/guidelines.xhtml

Table 1. Sample characteristics

Sample characteristic	**n=23**
Gender	
Male	5
Female	18
Age (years)	
40-49	7
50-59	4
60-69	7
70-74	5
Ethnicity	
Asian/ Asian British	22
Arab	1
IMD Decile	
1 (most deprived)-4	19
5-7	1
8-10 (least deprived)	3
Preferred language*	
English	17
Urdu	6

*An interpreter assisted with translation for participants who preferred to speak Urdu.

IMD, Index of Multiple Deprivation.

Three themes with sub-themes were produced and are outlined in Table 2.

Table 2. Summary of the themes and sub-themes identified in this study

Themes	Sub-themes
Trusted information as a foundation for engagement	Navigating health misinformation
	Trust in tailored advice
Making change feel manageable and worthwhile	Acknowledgement of everyday efforts and priorities
	The toll of guilt, regret, and rigid rules
	Working with cultural values
Creating environments where change feels possible	Safe, inclusive, and accessible environments

For peer review only - http://bmjopen.bmj.com/site/about/guidelines.xhtml

For peer review only

Trusted information as a foundation for engagement

This theme captures participants' need for clear and credible information, linking concerns about widespread misinformation with the desire for personalised advice from trusted sources.

Navigating health misinformation

Participants reported needing clear, trustworthy health guidance for their health behaviours. Many felt overwhelmed or paralysed by the sheer volume of conflicting advice, particularly when its credibility was uncertain or when they had difficulty securing GP appointments.

> *'Let's say you're trying to get healthy. There's so many people, like influencers that give you the wrong information… You feel overwhelmed. You just want to know the truth from someone.'* Nadia, Female, 44 years.

Information overload was not confined to the internet; contradictory tips exchanged among friends and family also amplified uncertainty.

> *'She doesn't follow online; she doesn't trust them at all… From her friends she gets suggestions like, 'Do not eat in the morning', 'Change your flour', 'Do some more walk', 'Do this, do that'—it's sometimes too much, and she's quite confused what she needs to do… That's why she wants two-three simple things from an expert that she could follow.'* Lubna via interpreter, Female, 59 years.

The lack of accessible, authoritative advice meant navigating multiple, often inconsistent information sources. In response, participants voiced a clear preference for brief, expert-endorsed guidance to bypass contradictory messages and give them a manageable starting point for behaviour change.

Trust in tailored advice

Participants valued that the information within the prototype carried the authority of a trusted institution and offered recommendations that felt specific to their own circumstances. Having a single,

For peer review only - http://bmjopen.bmj.com/site/about/guidelines.xhtml

reliable point of reference spared them the effort of searching online and turned uncertainty into a clear, personalised next step to act on.

> *'Some people just don't know what to do. Like you said, if you put on there [the app] guidelines, 'Try this, do this, this', you've got something to look at and then do it. Because if there's nothing to guide you… Nobody's going to look on YouTube or Google… But if it's on your app, your own health app, they'll say, 'Oh this is good for me, I'll try this'. Because it's all about you, and nobody else.'* Tahira, Female, 54 years.

Personalised advice mattered not only for its practicality but also for the relief it provided. When participants felt that guidance had been tailored to them, it meant not having to follow generic, one-size-fits-all guidance, which was sometimes experienced as overwhelming and detrimental to mental wellbeing.

> *'My doctor said, like,'Your cholesterol levels are very high'… I've tried to access information, just been given information sheets but no tailored advice or support in how we can manage or monitor better… you're trying to find information yourself, or tailor it to your way… mentally as well, it has an impact because you know the constant worry...'* Adeel, Male, 40 years.

Participants framed trusted, personalised guidance as both more credible and more emotionally manageable, giving them a clear direction in an otherwise confusing information landscape.

Making change feel manageable and worthwhile

This theme highlights participants' desire for advice relevant to their daily lives, while noting that unhelpful beliefs and cultural context could undermine its actionability if not addressed.

Acknowledgement of everyday efforts and priorities

Participants described how standard recommendations felt disconnected from their daily lives. They questioned whether informal and incidental forms of movement embedded in their daily routines counted towards meeting the physical activity recommendations.

For peer review only - http://bmjopen.bmj.com/site/about/guidelines.xhtml

'Even carrying the basket up and down the stairs… you don't realise it, how many times you go up and down.' Zara, Female, 41 years.

Recognising these small, everyday efforts could help motivate change and reduce discouragement, particularly when broader life pressures, like mental wellbeing, disabilities, cost of living, or caring responsibilities, shaped what felt possible to attempt.

'Especially like if they're looking after somebody elderly or they have kids or, you know, it's too far to go to the gym, it's too expensive, there's lots of barriers.' Sanjay, Male, 45 years.

Cultural traditions were an added layer of context shaping what felt manageable and sustainable. For example, foods shared during celebrations were often high in fat and sugar, making dietary changes feel incompatible with cultural norms and practices. Participants were open to learning culturally relevant alternatives to make healthy eating feel more realistic without having to abandon traditions.

The toll of guilt, regret, and rigid rules

The prospect of adopting healthier behaviours often carried emotional weight due to participants' beliefs about CVD risk and behaviour change. Some expressed fatalism, the belief that serious illness was inevitable regardless of current efforts. Others reflected on past choices with regret, expressing a sense that the damage to their health had already been done.

'If only we'd known all this when we were kids... we were eating all junk. But now... we realise, 'If only I had done that'.' Tahira, Female, 54 years.

Others described an all-or-nothing mindset, where a single lapse, such as eating unhealthy food, was seen as 'cheating' - a moral framing that imposed pressure and reinforced feelings of failure. Unhealthy eating was perceived to negate other efforts, such as exercise, despite cardiovascular benefits beyond weight loss.

For peer review only - http://bmjopen.bmj.com/site/about/guidelines.xhtml

> *'When I'm in any exercise session… in my head I'm telling myself I have to eat healthy. I can't cheat or anything. There's no point stretching if I'm going to go home and start eating rubbish.'* Mahila, Female, 65 years.

When prompted to discuss goal setting, participants often expressed scepticism. Setting goals was seen as overwhelming, particularly when goals were misaligned with current capabilities.

> *'You can't go from zero to 10 by goal setting. Sometimes it has the opposite effect for me… I prefer just doing natural things that feel comfortable for me… it's kind of like Slimming World, it creates a bit of like 'oh, am I a failure?'… Rather than if you just said 'Oh, you did 2000 last week, that's fantastic. Try to do 10 more'.'* Nadia, Female, 44 years.

Participants also described beliefs about diet and activity that made behaviour change feel unnecessarily difficult. Some of these reflected oversimplified or inaccurate understandings of what needed to be restricted.

> *'Chapati, rice — they make you fat.'* Rubina, Female, 74 years.

Such beliefs could lead to cognitively demanding restrictions that were taxing yet of limited benefit. However, more structured approaches, like calorie counting, which might theoretically reduce the need to eliminate foods entirely, were still overwhelming for some participants.

Working with cultural values

Although the prototype emphasised clinically recommended behaviours (e.g., physical activity, smoking cessation), participants raised culturally familiar practices, such as natural remedies. Although often raised tentatively for validation, these practices remained meaningful and motivating, offering a manageable starting point that could build momentum for further positive change.

> *'I found out this before what they said in the morning, if you drink warm water with a little bit of lemon in it… it makes your muscles cleaner, bigger, and then, you know, you wash it all down and then makes your muscles clean…. So last week I started doing the*

For peer review only - http://bmjopen.bmj.com/site/about/guidelines.xhtml

For peer review only

warm water with lemon in it, and I feel a lot better… Then you're thinking, oh, you did this in the morning. You don't want to ruin yourself..' Hafsa, Female, 65 years.

For some, health behaviours were framed as acts of gratitude or spiritual respect.

'She feels like our body is actually a gift from God, and we need to look after it, and she is being grateful by looking after it.' Farah via interpreter, Female, 47 years.

Aligning health messaging with these values can make behaviour change feel personally meaningful and rooted in religious and cultural identity. However, participants' sense of agency could be undermined if their efforts were overlooked. Some expressed disappointment or confusion when, despite avoiding smoking or alcohol, they still faced elevated CVD risk.

Creating environments where change feels possible

This theme captures how engagement depended on support feeling safe, inclusive, and accessible, with human connection providing the social encouragement and accountability needed to sustain change.

Safe, inclusive, and accessible environments

Participants described environmental and structural barriers that made behaviour change and accessing support feel out of reach. Concerns around affordability, discomfort, and safety varied but had a shared impact: they undermined confidence, increased the emotional cost of participation, and made healthy choices feel less viable, and at times even harmful.

While many participants valued outdoor activity for enjoyment and wellbeing, safety concerns could negate these benefits.

'Where we live, we suffer from racist abuse a lot... So you're very scared of stepping outside. You can't really switch off from 'Is someone behind me?', 'What is that person going to do?' It's not a stress-free walk - it's a stressful walk, but then it doesn't really do any good to the heart.' Rashida, Female, 63 years.

For peer review only - http://bmjopen.bmj.com/site/about/guidelines.xhtml

Affordability was another key barrier. Some participants joined subsidised programmes but stopped once funding ended.

> *'For 3 months [the exercise class] was free. Because they were not giving them any more vouchers, she said they asked for £25 per month. At that time, she couldn't pay, so that's why she stopped.'* Nargis via interpreter, Female, 64 years.

Expectations around appearance, body image, and other attendees also contributed to apprehension. Some participants avoided classes due to perceived dress codes.

> *'That was the reason why I was a bit apprehensive [to attend exercise classes], I thought 'Oh gosh, I'm going to have to go and buy a whole wardrobe of gym clothes.' But when we had our first session and I saw everyone in their normal clothes I thought 'OK, that's alright'.'* Rashida, Female, 63 years.

Others were initially worried about standing out as the least fit in the room but felt reassured once they saw a range of abilities among attendees. For some women, embarrassment about their bodies led them to prefer women-only spaces, which were valued for comfort and support.

Digital exclusion also limited access, with online booking systems proving a barrier for those lacking the skills or confidence to use them.

The motivating power of human connection

Participants stressed that support was more motivating when it involved social connection and human presence. Group settings and professional guidance reduced the burden of change, made healthy behaviours feel normal. and energised participants to act.

> *'Even a little bit of walking, it helps. But… I think what it is, it's a little bit of motivation. If one person's doing it, then people will copy… Because nobody wants to do it on their own. So there should be like groups.'* Tahira, Female, 65 years.

Even those favouring digital resources expressed that these often felt too passive without human involvement.

For peer review only - http://bmjopen.bmj.com/site/about/guidelines.xhtml

'There needs to be... some type of moderation, for example, if there's a health coach or someone working with the individual... Rather than just having information laid out. We have so many apps... but there's no one there telling me or pushing me that it's useful... then I feel like the person can lose track.' Adeel, Male, 40 years.

Cultural familiarity and representation amplified this effect. Professionals with the same ethnic background made advice feel more relevant and safer.

'It helps more, if it's Asian… the health advice is more better, when you're there there's more attention for you, you're more used to the South Asian people… It's like [a] comfortable environment for you.' Imran, Male, 44 years.

Some felt that rapport built through digital interactions could motivate them to attend in-person services. However, practical barriers such as work, childcare, and transport mean digital alternatives must also be designed to build a sense of human connection to support behaviour change.

Discussion

Key findings

Trusted, personalised information underpins engagement with health behaviour change, reflecting the need for clear, credible explanations that address misinformation and feel personally relevant. Behaviour change also needs to feel manageable and worthwhile through support that recognises everyday efforts, reframes unhelpful cognitions, offers realistic adaptations, and embraces cultural values. Safe, inclusive, accessible environments supported by human connection are essential, while policy and service-level action are required to address wider structural barriers.

Comparison with prior work

NHSHC advice is often generic and inconsistently followed up[5], leaving many, particularly those from socioeconomically deprived groups, without practical guidance. Whereas earlier NHSHC attendees reported being able to find the generic advice provided during NHSHCs elsewhere[24], our findings indicate that the current information landscape is contradictory and confusing. This challenge

For peer review only - http://bmjopen.bmj.com/site/about/guidelines.xhtml

is intensified for those with limited GP access to clarify guidance, disproportionately affecting South Asian populations since the shift to digital consultations[25]. Support must therefore help people navigate conflicting information by providing trusted, practical guidance that is personalised to their circumstances.

Consistent with prior work showing a preference for small, incremental changes[24], we found that acknowledging existing behaviours and tailoring recommendations matters. Participants wanted everyday activities, such as housework and walking, recognised as meaningful contributions. Overlooking these, or valued commitments like abstaining from smoking or alcohol, risks discouragement and undermining self-efficacy, a key factor in risk communication and behaviour change[26].

Fatalism has been highlighted as influential in health behaviour change[27]; our study shows how it can combine with regret, rigid rules, and unrealistic targets, to create significant cognitive and emotional strain. All-or-nothing mindsets can trigger guilt that discourages further effort, such as avoiding exercise because benefits beyond weight loss are not recognised. This highlights the need for support to counter unhelpful beliefs and reduce the emotional toll of change by reframing it as a process of learning and adaptation rather than strict adherence to rules. Wider social factors, such as racism, further reduced capacity for self-regulation and compounded these challenges.

Earlier studies also noted that some perspectives within minoritised ethnic groups diverge from the biomedical model of health[28,29]. Our findings support this, and identify ways support can leverage cultural beliefs and ensure acceptability. For example, recognising culturally congruent practices while sensitively explaining other risk factors may reduce feelings of unfairness, preserve self-efficacy, and harness cultural values as a driver for change.

Calls for greater representation of minoritised ethnic groups in NHSHC research have highlighted the need to understand how ethnicity and deprivation intersect to shape access and engagement[30]. We addressed this by recruiting adults from minoritised ethnic backgrounds in high-deprivation areas to explore support needs and preferences. The relevance of our findings is reinforced by evidence from

For peer review only - http://bmjopen.bmj.com/site/about/guidelines.xhtml

other health contexts, such as Iqbal, et al.[31], who report similar barriers, including limited culturally relevant health advice and safe opportunities for physical activity among pregnant British-Pakistani women. These shared barriers indicate systemic issues, pointing towards the need for both system-level change and tailored support.

Strengths and limitations

A key strength of this study was the recruitment of predominantly South Asian women living in deprived communities, a group rarely represented in research despite their heightened risk[32] and significant influence on household health practices such as cooking[33]. Using a digital prototype emulated the process of receiving CVD risk feedback and support in a format similar to the digital NHSHC pilot[9]. The realistic format helped participants articulate useful support strategies relevant to both face-to-face and digital delivery. Although the prototype was configured only to signpost to weight-loss services, we encouraged participants to consider how the same approach would apply to any behaviour or support need. Nonetheless, this may have limited responses from those with different health priorities. While the use of hypothetical CVD risk information could be argued to have influenced participants' responses, including the amount or intensity of support they felt they needed, this is unlikely to have affected our findings, as our focus was on the qualities of behaviour change support preferred, which are unlikely to depend on a specific risk level. A further limitation is that recruitment from a single area limits transferability and may not reflect the support needs and preferences of other minoritised or socioeconomically deprived communities.

Implications

This study adds to the evidence that preventative strategies must be co-designed to be trusted, culturally relevant, and embedded in everyday contexts[8]. For NHSHCs, this means moving beyond generic advice to provide guidance that is personally relevant, credible, and responsive to the priorities of underserved groups.

The availability of referral pathways remains important[34,35], but engagement also depends on whether services feel safe, inclusive, and practically accessible. Both real concerns (e.g. racism, exclusion) and

For peer review only - http://bmjopen.bmj.com/site/about/guidelines.xhtml

perceived barriers (e.g. assumptions about gym clothing) influence willingness to engage. Commissioners and providers therefore need to prioritise creating supportive environments that feel welcoming, so NHSHCs can function more effectively as gateways to follow-up support and improved health outcomes[36].

Our findings have important implications for the ongoing development of the digital NHSHC. Evaluation of the pilot identified that some users found the digital signposting process, which directs individuals to follow-up support based on their selected health priorities and perceived barriers, too impersonal[37]. We found that incorporating human support into the digital pathway could help build the rapport that some participants felt they would need to engage with follow-up services. Without this opportunity for discussion, the digital pathway depends on users self-reporting their behaviour change priorities and barriers[37], which limits opportunities to explore or clarify support needs and, in turn, may reduce engagement with appropriate follow-up support. Incorporating an option for human support into the digital NHSHC could help address this limitation and improve the effectiveness of behaviour change signposting.

Future strategies should build on these findings as a foundation for the co-design and evaluation of behaviour change support for minoritised ethnic groups in deprived areas. Priorities include integrating everyday practices and cultural commitments, addressing unhelpful beliefs about CVD prevention, and improving perceptions of support services, while ensuring that follow-up options are safe and inclusive. Alongside policy changes to remove structural barriers, such as cost, these approaches are critical to making the NHSHC programme more equitable and effective for underserved groups. Future research could synthesise findings across multiple studies and map support needs to frameworks such as Capability, Opportunity, Motivation - Behaviour (COM-B)[38] to identify the factors influencing engagement with behaviour change support as part of the NHSHC among underserved populations, providing a foundation for designing targeted interventions.

For peer review only - http://bmjopen.bmj.com/site/about/guidelines.xhtml

Conclusion

NHSHC behaviour change support for minoritised ethnic adults in deprived areas needs to be trusted, culturally relevant, and accessible, with advice grounded in everyday contexts and values. Embedding these principles can improve engagement, strengthen the programme’s role as a gateway to follow-up support, and reduce inequalities in CVD prevention.

For peer review only

For peer review only - http://bmjopen.bmj.com/site/about/guidelines.xhtml

References

1. World Health Organisation. Cardiovascular diseases (CVDs), 2021. Available: https://www.who.int/news-room/fact-sheets/detail/cardiovascular-diseases-(cvds).

2. British Heart Foundation. Early heart disease deaths rise to 14-year high, 2024. Available: https://www.bhf.org.uk/what-we-do/news-from-the-bhf/news-archive/2024/january/early-heart-disease-deaths-rise-to-14-year-high

3. Public Health England. Health matters: preventing cardiovascular disease, 2019. Available: https://www.gov.uk/government/publications/health-matters-preventing-cardiovascular-disease/health-matters-preventing-cardiovascular-disease

4. Usher-Smith JA, Mant J, Martin A, et al. *NHS health check programme rapid evidence synthesis.* University of Cambridge, 2017.

5. Riley R, Coghill N, Montgomery A, Feder G, Horwood J. The provision of NHS health checks in a community setting: an ethnographic account. *BMC Health Services Research*. 2015;15(1):546. doi: 10.1186/s12913-015-1209-1

6. Corlett SA, Krska J. Evaluation of NHS Health Checks provided by community pharmacies. *Journal of Public Health*. 2015;38(4):e516-e23. doi: 10.1093/pubmed/fdv153

7. Usher-Smith J, A., Harte E, MacLure C, et al. Patient experience of NHS health checks: a systematic review and qualitative synthesis. *BMJ Open*. 2017;7(8):e017169. doi: 10.1136/bmjopen-2017-017169

8. Grice-Jackson T, Rogers I, Ford E, et al. The pre-implementation phase of a project seeking to deliver a community-based CVD prevention intervention (spices-sussex): a qualitative study exploring views and experience relating to intervention development. *Health Promotion Practice.* 2024;25(6):1009-22. doi: 10.1177/15248399231182139

9. Salway R, Sillero-Rejon C, Forte C, et al. A service evaluation of the uptake and effectiveness of a digital delivery of the NHS health check service. *BMJ Open*. 2024;14(11):e091417. doi: 10.1136/bmjopen-2024-091417

For peer review only - http://bmjopen.bmj.com/site/about/guidelines.xhtml

10. Malterud K, Siersma VD, Guassora AD. Sample size in qualitative interview studies: guided by information power. *Qualitative Health Research*. 2016;26(13):1753-60. doi: 10.1177/1049732315617444

11. University of Manchester. Digital health software and platforms, 2024. Available: https://sites.manchester.ac.uk/digital-health-software-and-platforms/

12. Marvel, 2024. Available: https://marvelapp.com/

13. Witteman HO, Fuhrel-Forbis A, Wijeysundera HC, et al. Animated randomness, avatars, movement, and personalization in risk graphics. *Journal of Medical Internet Research.* 2014;16(3):e80. doi: 10.2196/jmir.2895

14. Cowap L, Riley V, Grogan S, et al. "They are saying it's high, but I think it's quite low": exploring cardiovascular disease risk communication in NHS health checks through video-stimulated recall interviews with patients – a qualitative study. *BMC Primary Care.* 2024;25(1) doi: 10.1186/s12875-024-02357-w

15. Schulberg SD, Ferry AV, Jin K, et al. Cardiovascular risk communication strategies in primary prevention. A systematic review with narrative synthesis. *Journal of Advanced Nursing.* 2022;78(10):3116-40. doi: 10.1111/jan.15327

16. Rogers RW. Cognitive and Physiological Processes in Fear Appeals and Attitude Change: A Revised Theory of Protection Motivation. In: Cacioppo J, Petty R, eds. *Social Psychophysiology*. New York: Guilford.1983.

17. Leventhal H, Benyamini Y, Brownlee S, et al. Illness Representations: Theoretical Foundations. In: Petrie KJ, Weinman JA, eds. *Perceptions of Health and Illness*. Amsterdam: Harwood Academic Press 1997:19-45.

18. Pate A, Jiang B, Huang Y-T, et al. CHARIOT: development and internal validation of a cardiovascular health assessment and risk-based intervention optimisation tool. *medRxiv*. 2025;2025.09.18.25336064. doi:10.1101/2025.09.18.25336064

19. McMillan B, Fox S, Lyons M, et al. Using patient and public involvement to improve the research design and funding application for a project aimed at fostering a more collaborative approach

For peer review only - http://bmjopen.bmj.com/site/about/guidelines.xhtml

to the NHS Health Check: the CaVIAR project (Better Care Via Improved Access to Records). *Res Involv Engagem.* 2018;4:18. doi: 10.1186/s40900-018-0101-7.

20. Braun V, Clarke V. Reflecting on reflexive thematic analysis. *Qualitative Research in Sport, Exercise and Health.* 2019;11(4):589-97. doi: 10.1080/2159676X.2019.1628806

21. Braun, V, Clarke, V. Toward good practice in thematic analysis: Avoiding common problems and be(com)ing a knowing researcher. *International Journal of Transgender Health*. 2023;24(1):1–6. doi: 10.1080/26895269.2022.2129597.

22. O'Brien BC, Harris IB, Beckman TJ, Reed DA, Cook DA. Standards for reporting qualitative research: A synthesis of recommendations. *Academic Medicine*. 2014;89(9):1245–51. Available: https://journals.lww.com/academicmedicine/fulltext/2014/09000/Standards_for_Reporting_Qualitative_Research__A.21.aspx

23. O'Brien BC, Harris IB, Beckman TJ, Reed DA, Cook DA. The SRQR reporting checklist. In: Harwood J, Albury C, Beyer J de, Schlüssel M, Collins G, eds. *The EQUATOR network reporting guideline platform.* The UK EQUATOR Centre 2025. Available: https:/resources.equator-network.org/reporting-guidelines/srqr/srqr-checklist.docx

24. McNaughton RJ, Shucksmith J. Reasons for (non)compliance with intervention following identification of 'high-risk' status in the NHS Health Check programme. *Journal of Public Health.* 2015;37(2):218-25. doi: 10.1093/pubmed/fdu066

25. Small N, Masood Y, Stevenson F, et al. Exploring the experiences and preferences of South Asian patients' of primary care in England since COVID-19. *Health Expectations.* 2024;27(1) doi: 10.1111/hex.13982

26. French DP, Cameron E, Benton JS, et al. Can communicating personalised disease risk promote healthy behaviour change? a systematic review of systematic reviews. *Annals of Behavioral Medicine.* 2017;51(5):718. doi: 10.1007/s12160-017-9895-z

27. Honey S, Hill K, Murray J, et al. Patients' responses to the communication of vascular risk in primary care: a qualitative study. *Primary Health Care Research & Development.* 2015;16(1):61-70.

For peer review only - http://bmjopen.bmj.com/site/about/guidelines.xhtml

28. Hawking MKD, Timmis A, Wilkins F, et al. Improving cardiovascular disease risk communication in NHS Health Checks: a qualitative study. *BMJ Open.* 2019;9(8):e026058. doi: 10.1136/bmjopen-2018-026058

29. Riley R, Coghill N, Montgomery A, et al. The provision of NHS health checks in a community setting: an ethnographic account. *BMC Health Services Research.* 2015;15(1):546. doi: 10.1186/s12913-015-1209-1

30. Eberhardt J, Kane L, Portman R, et al. Barriers and facilitators of NHS health checks in socioeconomically deprived communities in the north east of england: a qualitative study with peer researchers. *Health Expectations.* 2025;28(2) doi: 10.1111/hex.70199

31. Iqbal H, West J, McEachan RR, Haith-Cooper M. Identifying the health concerns of pregnant british pakistani women living in deprived areas: a qualitative study. *Maternal and Child Health Journal.* 2024;28(3):489-95.

32. Redwood S, Gill PS. Under-representation of minority ethnic groups in research — call for action. *British Journal of General Practice.* 2013;63(612):342-43. doi: 10.3399/bjgp13x668456

33. Vallianatos H, Raine K. Consuming food and constructing identities among arabic and south asian immigrant women. *Food, Culture & Society.* 2008;11(3):355-73. doi: 10.2752/175174408X347900

34. Alageel S, Gulliford MC, McDermott L, Wright AJ. Implementing multiple health behaviour change interventions for cardiovascular risk reduction in primary care: a qualitative study. *BMC Family Practice.* 2018;19(1):171. doi: 10.1186/s12875-018-0860-0

35. Gadsby EW, Krska J, Duddy C, et al. The NHS Health Check programme: a survey of programme delivery in England before and after the Covid-19 pandemic response. *NIHR Open Research.* 2023;3:32. doi: 10.3310/nihropenres.13436.2

36. Duddy C, Gadsby E, Hibberd V, et al. What happens after an NHS Health Check? A survey and realist review. *Health and Social Care Delivery Research.* 2022;11(12). doi: 10.3310/RGTH4127

For peer review only - http://bmjopen.bmj.com/site/about/guidelines.xhtml

37. Forte C, Grey EB, Jessiman P, et al. Exploring service users' and healthcare professionals' experience of digital and face-to-face Health Checks in England: a qualitative study. *BMJ Open.* 2025;15(3):e090492. doi: 10.1136/ bmjopen-2024-090492

38. Michie S, Van Stralen MM, West R. The behaviour change wheel: A new method for characterising and designing behaviour change interventions. *Implementation Science*. 2011;6(1):42. doi: 10.1186/1748-5908-6-42

For peer review only

For peer review only - http://bmjopen.bmj.com/site/about/guidelines.xhtml

Declarations

Acknowledgements

We thank the staff of the community organisation for their assistance with recruitment and translation, and the community members who participated for their invaluable contributions to this research. We also extend our gratitude to the PPI members for their important input.

Contributors

SG, the guarantor, led the study design and data collection, coded and analysed the data, and drafted the manuscript. YKB, DPF, and BM contributed to the conceptualisation, supervised coding and analysis, and reviewed the manuscript. All authors read and approved the final manuscript.

Funding

This research was funded by the EMIS National User Group (https://emisnug.org.uk/). The algorithm development was funded by the National Institute for Health Research (NIHR) School for Primary Care Research (SPCR) (reference: NIHR SPCR-2021-2026, grant number 648) and Endeavour Health Charitable Trust. The views expressed are those of the authors and not necessarily those of the NIHR, the Department of Health and Social Care, Endeavour Health, or the EMIS NUG.

Competing interests

The authors declare no competing interests.

Patient and public involvement

Patients and/or the public were involved in the design of this research. See the Methods section for further details.

Ethics approval

This study received University of Manchester Research Ethics approval (REF 2024-19916-37529). All participants provided written or electronic informed consent.

For peer review only

For peer review only - http://bmjopen.bmj.com/site/about/guidelines.xhtml

Data availability statement

The data from this study are anonymised but contain context-specific narratives that could risk participant identification; to maintain confidentiality, they are not publicly available and cannot be shared.

For peer review only

Figure 1. Screenshots of the digital prototype presenting CVD risk information and signposting to behaviour change support.

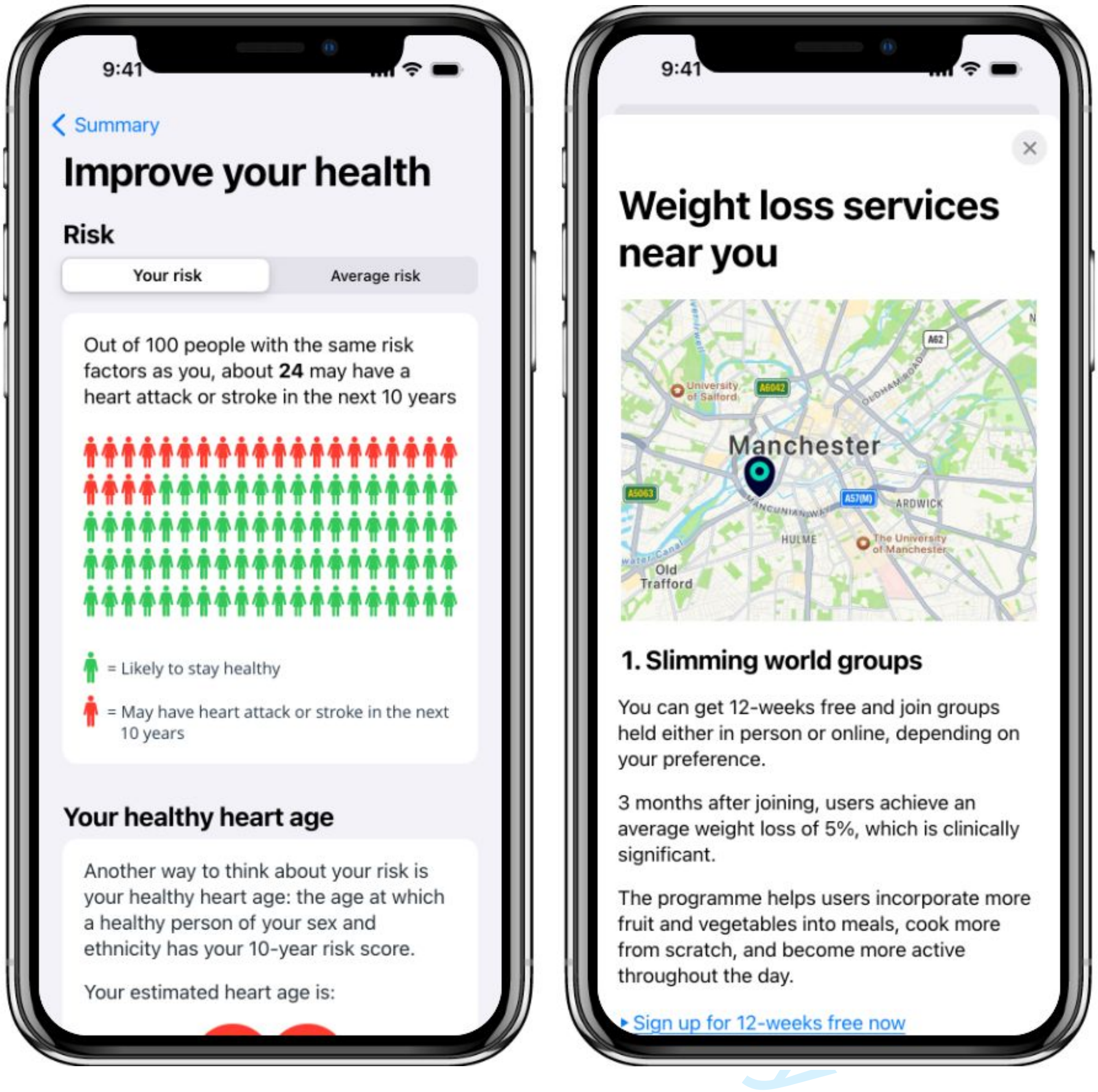

The first screen (left) shows the cardiovascular risk result for a hypothetical user. The second screen (right) shows signposting to publicly available local organisations (e.g., Slimming World).

Supplementary File

Supplementary Material S1: Screenshots of the Digital Prototype (Final Version)

Note: The prototype signposts to real organisations (e.g., Slimming World), which are publicly available. All personal details and cardiovascular risk information shown in this prototype are fictitious; no real participant data were used.

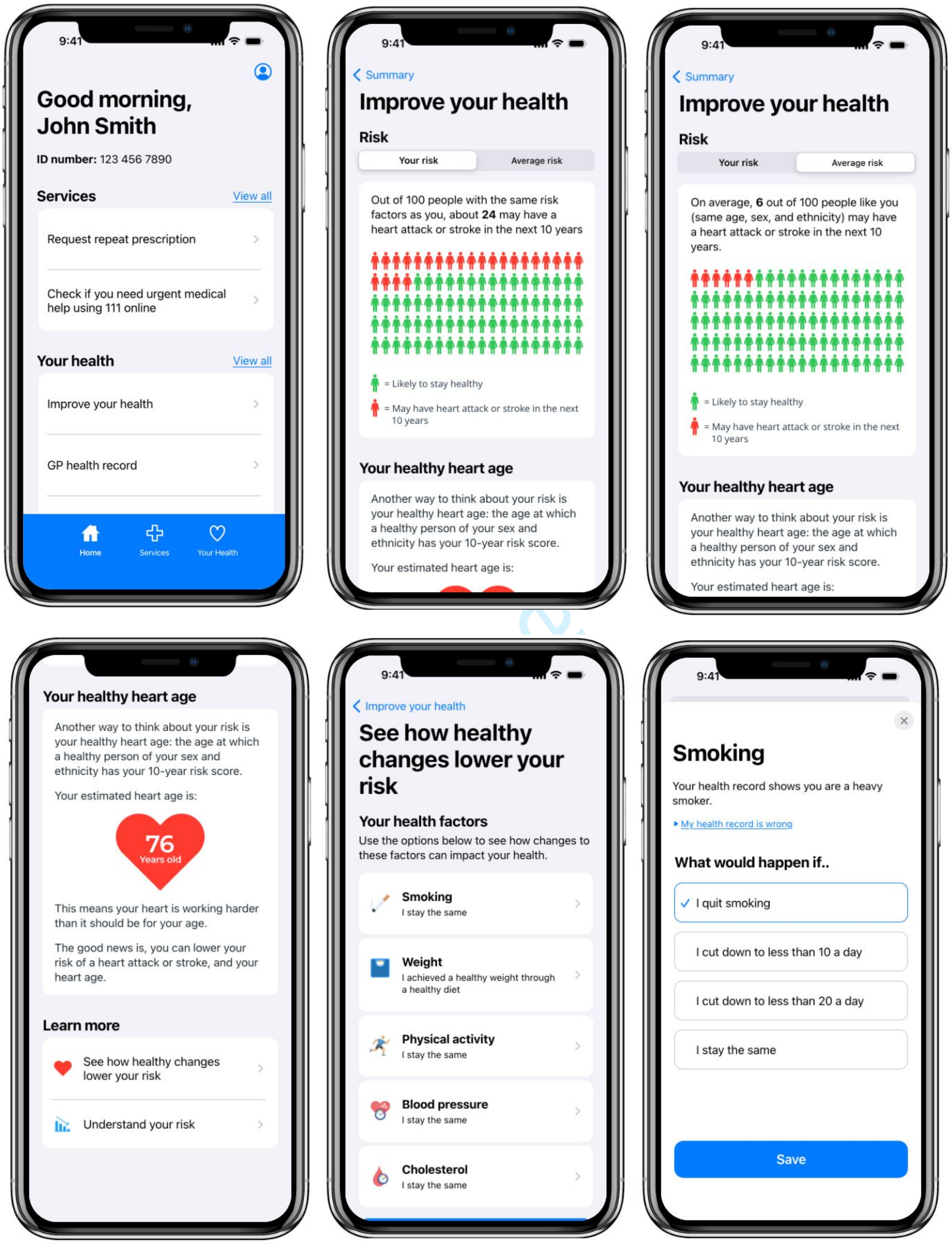

1

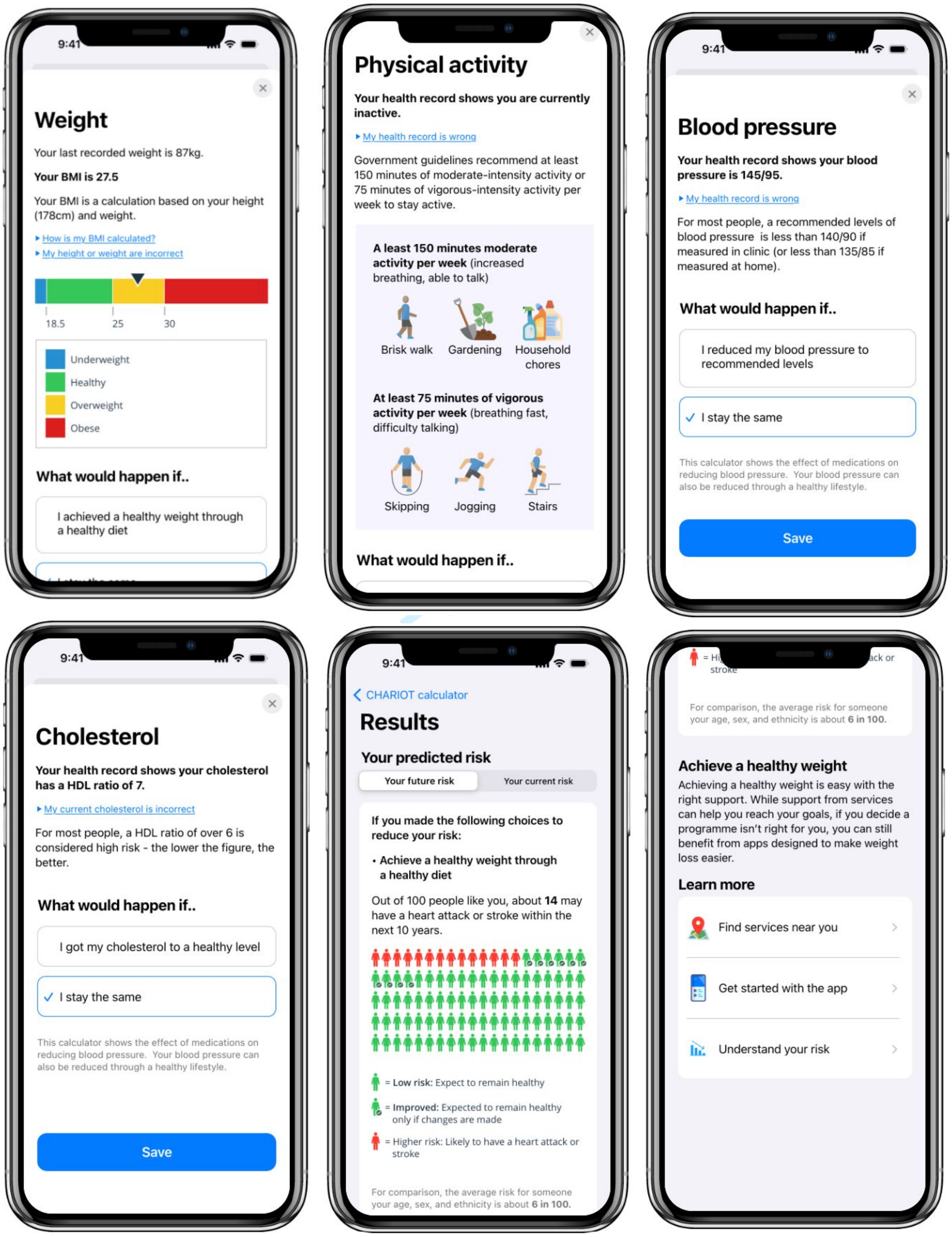
9:41
Weight
Your last recorded weight is 87kg.
Your BMI is 27.5
Your BMI is a calculation based on your height (178cm) and weight.
How is my BMI calculated?
My height or weight are incorrect
18.5
25
30
Underweight
Healthy
Overweight
Obese
What would happen if..
I achieved a healthy weight through a healthy diet
Physical activity
Your health record shows you are currently inactive.
My health record is wrong
Government guidelines recommend at least 150 minutes of moderate-intensity activity or 75 minutes of vigorous-intensity activity per week to stay active.
A least 150 minutes moderate activity per week (increased breathing, able to talk)
Brisk walk
Gardening
Household chores
At least 75 minutes of vigorous activity per week (breathing fast, difficulty talking)
Skipping
Jogging
Stairs
What would happen if..
9:41
Blood pressure
Your health record shows your blood pressure is 145/95.
My health record is wrong
For most people, a recommended levels of blood pressure is less than 140/90 if measured in clinic (or less than 135/85 if measured at home).
What would happen if..
I reduced my blood pressure to recommended levels
I stay the same
This calculator shows the effect of medications on reducing blood pressure. Your blood pressure can also be reduced through a healthy lifestyle.
Save
9:41
Cholesterol
Your health record shows your cholesterol has a HDL ratio of 7.
My current cholesterol is incorrect
For most people, a HDL ratio of over 6 is considered high risk - the lower the figure, the better.
What would happen if..
I got my cholesterol to a healthy level
I stay the same
This calculator shows the effect of medications on reducing blood pressure. Your blood pressure can also be reduced through a healthy lifestyle.
Save
9:41
CHARIOT calculator
Results
Your predicted risk
Your future risk
Your current risk
If you made the following choices to reduce your risk:
• Achieve a healthy weight through a healthy diet
Out of 100 people like you, about 14 may have a heart attack or stroke within the next 10 years.
= Low risk: Expect to remain healthy
= Improved: Expected to remain healthy only if changes are made
= Higher risk: Likely to have a heart attack or stroke
For comparison, the average risk for someone your age, sex, and ethnicity is about 6 in 100.
For comparison, the average risk for someone your age, sex, and ethnicity is about 6 in 100.
Achieve a healthy weight
Achieving a healthy weight is easy with the right support. While support from services can help you reach your goals, if you decide a programme isn't right for you, you can still benefit from apps designed to make weight loss easier.
Learn more
Find services near you
Get started with the app
Understand your risk

2

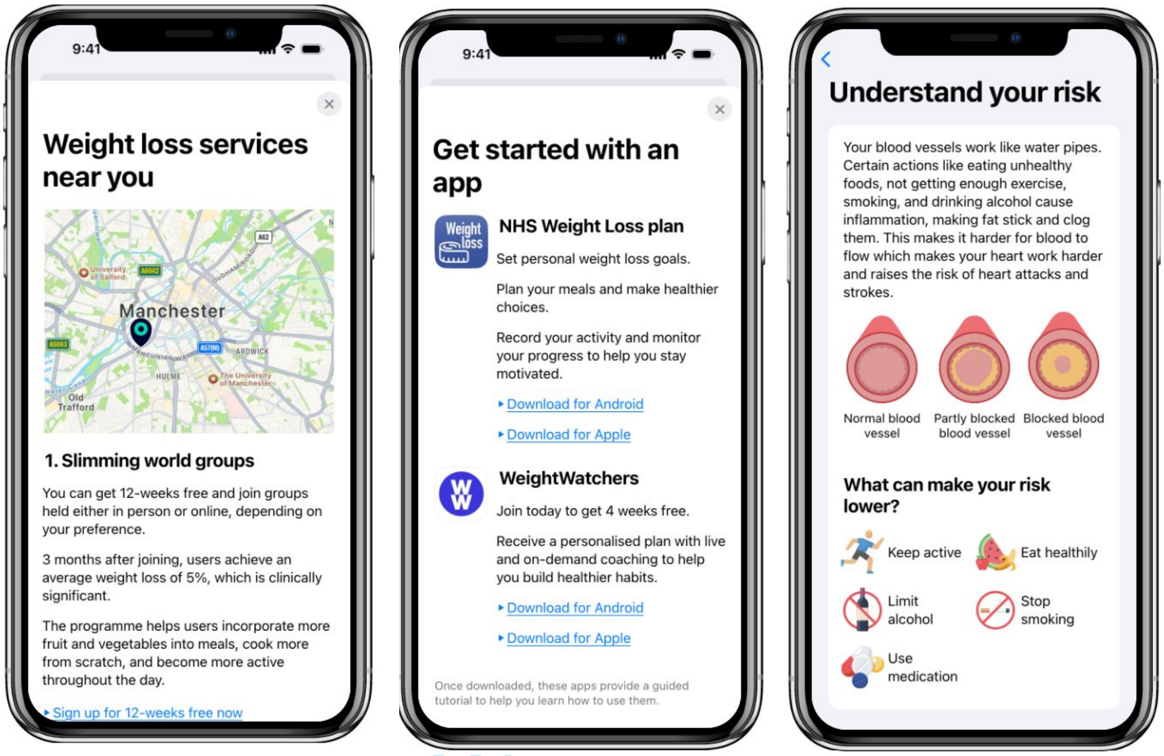

For peer review only - http://bmjopen.bmj.com/site/about/guidelines.xhtml

Supplementary Material S2: Focus Group and Interview Topic Guides

These topic guides were used across the wider qualitative study. Not all questions contributed data to the analysis reported in the accompanying manuscript; only the relevant sections of the dataset were analysed for the manuscript's specific aims.

1. Focus group/ workshop schedule

Introduction and housekeeping

- Welcome participants, thank them for attending, explain ground rules.
- Explain the purpose of the session and its role within the wider study.
- Introduce the research team and invite participants to introduce themselves.
- Emphasise that all contributions are confidential and that participants should respect each other's privacy.
- Explain that all data will be anonymised during transcription and reporting; no identifiable information will be included in publications.
- Explore participants' existing understanding of cardiovascular disease (CVD) and familiarity with NHS Health Checks before explaining context for the prototype.
- Show the following prototype presentation (1.1-1.7).

Prototype presentation

1.1 Navigation page

- A discussion about how to navigate to the calculator
- Consider what language/labels feel clear and accessible.

1.2 Text explanation of risk calculator

- A discussion about whether people understand text information explaining risk calculator, and if any other information is needed to support their understanding.

1.3 Risk score result and visual display

- A discussion around presentation of risk results, including icon arrays, colours used.

1.4 Heart age result display

For peer review only - http://bmjopen.bmj.com/site/about/guidelines.xhtml

- A discussion about presentation of Heart Age and comparative risks.
- Explore whether participants find this combination of information helpful for understanding cardiovascular risk.

1.5 Risk explanation

- Gather views on how risk information is presented, if it is understood, and views on the analogy used.
- Discuss what would make the information most understandable and meaningful.

1.6 Risk manipulation

- Explore views on seeing potential reductions in risk through health behaiour changes (stopping smoking, losing weight, increasing physical activity, reducing blood pressure, improving cholesterol).
- Discuss whether the behaviour change options presented are perceived as feasible or motivating.
- Explore how participants would like reductions in risk to be presented (use of risk bar, icon array, and whether the green icons with ticks used to show the difference between current and potential risk are clear).

1.7 Behaviour change support signposting

- Explore views on the example of how a hypothetical user could be signposted to resources to help them change CVD risk factors (e.g. smoking cessation services, weight loss services, information on how they could reduce their blood pressure) etc.
- Explore views on how helpful this concept would be, what could be improved/ made more clear or useful.

Closing discussion

- Invite participants to share any additional thoughts or reflections.
- Thank participants for their contributions.
- Remind participants they can contact the research team if they have follow-up questions or concerns.

For peer review only - http://bmjopen.bmj.com/site/about/guidelines.xhtml

2. Interview Topic Guide

1. Was there anything about the risk score or information that felt confusing or unclear to you?
2. What, if anything, was memorable?

Prompt: Are there any particular features you think would encourage you to use this app?

3. At the beginning it tells you your risk score. Would seeing your risk presented like this influence you in any way, in terms of making healthier changes?
4. Do you think you would like to look back at your risk score over time, to see how it changes if you went away and made healthier changes?
5. Could you see yourself using this tool independently, or do you think you'd need to be shown it by a professional first?
6. Is there anything other features you would like to be included, that would make the app more appealing?

Prompt: How would you feel about there being techniques to help you manage making healthier changes, e.g., goal setting?

7. Is there anything that isn't on there that you would have liked to learn about, related to heart health?
8. Did you leave with the impression from the app that healthier changes could make a difference to heart health?

Prompt: or cholesterol, blood pressure?

4. There was an example of how grease builds up in pipes used to explain what goes on inside your blood vessels- how did you find that?

Prompt: How did it make you feel towards making healthy or unhealthy choices?

10. If you were thinking about improving your heart health, are there any challenges that come to mind that the app, or other support, might be able to help with?

Prompt: Are there any other ways you'd prefer to see this information, e.g., videos?

If videos: Would you be interested in seeing animations or real people in videos?

For peer review only - http://bmjopen.bmj.com/site/about/guidelines.xhtml

5. There were weight loss programmes listed on the app as options for support. The idea would be the same for any other goal that people wanted to achieve with their health where that kind of support is available. Do you think you would make the leap to signing up to programmes like that to receive support?

12. Is there anything about a programme that would make going more or less appealing to you? *Prompts: Who runs it, what age range it is targeted for.*

13. Are you more interested doing things in the community in person, or would you access support online – why?

14. How would you describe your confidence in terms of going on to make healthier changes after receiving this kind of information?

Prompt: What kind of support would help you feel more confident about making and sticking to healthier changes?

15. Is there anything else you wanted to add - any final thoughts?

For peer review only - http://bmjopen.bmj.com/site/about/guidelines.xhtml

Supplementary Material Table S3: SRQR Checklist

Standards for Reporting Qualitative Research (SRQR): a synthesis of recommendations

All items of this checklist are directly cited from O'Brien BC, Harris IB, Beckman TJ, Reed DA, Cook DA.

Standards for reporting qualitative research: A synthesis of recommendations. *Academic Medicine*.

2014;89(9):1245-1251. doi:10.1097/ACM.0000000000000388

	Item Description	Location (or reason for not reporting)
**Title & Abstract**		
Title	Describe the nature and topic of the study. Identify the study as qualitative or indicate the approach or data collection methods.	Title page
Abstract	Summarise the key elements of the study using the abstract format of the intended publication.	Title page
**Introduction**		
Problem Formulation	Describe the problem/phenomenon studied, its significance, relevant theory and empirical work, and gaps in current knowledge.	Introduction; paragraphs 1, 2, 3, 4
Purpose or research question	Describe the purpose of the study and specific objectives or questions.	Introduction; paragraph 4. Aim
**Methods**		
Qualitative approach and research paradigm	Describe your qualitative approach, your guiding theory (if appropriate), and research paradigm, and reasons for your choices.	Methods, Study design, Analysis
Researcher characteristics and reflexivity	Describe how researchers' characteristics may influence the research, including personal attributes, qualifications/experience, relationship with participants, assumptions, and/or presuppositions; potential or actual interaction between researchers' characteristics and the research questions, approach, methods, results and/or transferability.	Methods, Analysis; paragraph 2
Context	Describe the setting/site(s) in which the study was conducted, why it was selected, and any other salient contextual factors that may influence the study.	Methods, Participant recruitment; paragraph 1
Sampling strategy	Describe how and why research participants, documents, or events were selected; criteria for deciding when no further sampling was necessary, and the rationale for those criteria.	Methods, Participant recruitment; paragraph 2
Ethical issues pertaining to human subjects	Describe any approval by an appropriate ethics review board and participant consent, or explain any lack thereof. Describe any other confidentiality and data security issues.	Methods, Study design
Data collection methods	Describe the types of data collected; details of data collection procedures including (as appropriate) start and stop dates of data collection and analysis, iterative process, triangulation of sources/methods, and modification of procedures in response to evolving study findings. Describe your rationale for these choices.	Methods, Data collection

For peer review only - http://bmjopen.bmj.com/site/about/guidelines.xhtml

Data collection instruments and technologies	Describe any instruments (e.g., interview guides, questionnaires) and devices (e.g., audio recorders) used for data collection; describe if/how the instrument(s) changed over the course of the study.	Methods, Materials
Units of study	Describe the number and relevant characteristics of participants, documents, or events included in the study. Describe the level of participation.	Results; paragraph , Table 1
Data processing	Describe the methods for processing data prior to and during analysis, including transcription, data entry, data management and security, verification of data integrity, data coding, and anonymisation / deidentification of excerpts.	Methods, Analysis; paragraph 1
Data analysis	Describe the process by which inferences, themes, etc. were identified and developed, including the researchers involved in data analysis; usually references a specific paradigm or approach. Describe why you chose this process.	Methods, Analysis; paragraph 1, 2
Techniques to enhance trustworthiness	Describe any techniques to enhance trustworthiness and credibility of data analysis,(e.g., member checking, triangulation, audit trail). Describe why you chose these techniques.	Methods, Analysis; paragraph 2
**Results**		
Synthesis and interpretation	Describe the main findings (e.g., interpretations, inferences, and themes); might include development of a theory or model, or integration with prior research or theory.	Results; Table 2. & First paragraph beneath each theme heading.
Links to empirical data	Provide evidence (e.g., quotes, field notes, text excerpts, photographs) to substantiate analytic findings.	Results; embedded throughout (e.g., paragraph 4).
**Discussion**		
Integration with prior work, implications, transferability, and contribution(s) to the field	Summarize the main findings, explain how findings and conclusions connect to, support, elaborate on, or challenge conclusions of earlier scholarship; discuss the scope of application/generalizability; identify unique contribution(s) to scholarship in a discipline or field.	Discussion; paragraph 1-10
Limitations	Discuss the trustworthiness and limitations of findings	Discussion; paragraph 7
**Other**		
Conflicts of interest	Describe any potential sources of influence or perceived influence on study conduct and conclusions. Describe how these were managed.	Declarations, Competing interests
Funding	Describe sources of funding and other support. Describe the role of funders in data collection, interpretation, and reporting.	Declarations, Funding

For peer review only - http://bmjopen.bmj.com/site/about/guidelines.xhtml
